# The Rise of SARS-CoV-2 Variants and the Role of Convalescent Plasma Therapy for Management of Infections

**DOI:** 10.3390/life11080734

**Published:** 2021-07-23

**Authors:** Mohamed Moubarak, Keneth Iceland Kasozi, Helal F. Hetta, Hazem M. Shaheen, Abdur Rauf, Hayder M. Al-kuraishy, Safaa Qusti, Eida M. Alshammari, Emmanuel Tiyo Ayikobua, Fred Ssempijja, Adam Moyosore Afodun, Ritah Kenganzi, Ibe Michael Usman, Juma John Ochieng, Lawrence Obado Osuwat, Kevin Matama, Ali I. Al-Gareeb, Emmanuel Kairania, Monica Musenero, Susan Christina Welburn, Gaber El-Saber Batiha

**Affiliations:** 1 Department of Pharmacology and Therapeutics, Faculty of Veterinary Medicine, Damanhour University, Damanhour 22511, Egypt; hmoubarak460@gmail.com (M.M.); dr_hazemshaheen3010@yahoo.com (H.M.S.); 2 Infection Medicine, Deanery of Biomedical Sciences, College of Medicine and Veterinary Medicine, The University of Edinburgh, 1 George Square, Edinburgh EH8 9JZ, UK; 3 School of Medicine, Kabale University, Kabale P.O. Box 317, Uganda; 4 Department of Medical Microbiology and Immunology, Faculty of Medicine, Assiut University, Assiut 71515, Egypt; helalhetta@aun.edu.eg; 5 Department of Chemistry, University of Swabi, Swabi 23561, Pakistan; mashaljcs@yahoo.com; 6 Department of Clinical Pharmacology and Medicine, College of Medicine, Al-Mustansiriyia University, P.O. Box 14022 Baghdad, Iraq; Hayderm36@yahoo.com; 7 Biochemistry Department, Faculty of Science, King Abdulaziz University, Jeddah 21589, Saudi Arabia; squsti@kau.edu.sa; 8 Department of Chemistry, College of Sciences, University of Ha’il, Ha’il 2440, Saudi Arabia; eida.alshammari@uoh.edu.sa; 9 School of Health Sciences, Soroti University, Soroti P.O. Box 211, Uganda; tiyosbase@gmail.com (E.T.A.); longodia@gmail.com (L.O.O.); 10 Department of Anatomy, Faculty of Biomedical Sciences, Kampala International University, Western Campus, Bushenyi P.O. Box 71, Uganda; kalanzifr@yahoo.com (F.S.); gopama13@gmail.com (I.M.U.); john.juma@kiu.ac.ug (J.J.O.); 11 Department of Anatomy and Cell Biology, Faculty of Health Sciences, Busitema University, Tororo P.O. Box 236, Uganda; afodunadam@yahoo.com (A.M.A.); kairaniaemma@gmail.com (E.K.); 12 Department of Medical Laboratory Sciences, School of Allied Health Sciences, Kampala International University Teaching Hospital, Bushenyi P.O. Box 71, Uganda; kenganziritah@gmail.com; 13 School of Pharmacy, Kampala International University, Western Campus, Bushenyi P.O. Box 71, Uganda; kevicematama@gmail.com; 14 Department of Pharmacology, Toxicology and Medicine, College of Medicine Al-Mustansiriya University, Baghdad P.O. Box 14022, Iraq; dr.alialgareeb78@yahoo.com; 15 Ministry of Science Technology and Innovations, Government of Uganda, Kampala P.O. Box 7466, Uganda; mmusenero@gmail.com; 16 Zhejiang University-University of Edinburgh Joint Institute, Zhejiang University, International Campus, 718 East Haizhou Road, Haining 314400, China

**Keywords:** coronavirus, COVID-19 therapy, COVID-19 vaccine, COVID-19 convalescent therapy, SARS-CoV-2 infection, human antibodies, variants of concern, Ebola

## Abstract

Novel therapies for the treatment of COVID-19 are continuing to emerge as the SARS-Cov-2 pandemic progresses. PCR remains the standard benchmark for initial diagnosis of COVID-19 infection, while advances in immunological profiling are guiding clinical treatment. The SARS-Cov-2 virus has undergone multiple mutations since its emergence in 2019, resulting in changes in virulence that have impacted on disease severity globally. The emergence of more virulent variants of SARS-Cov-2 remains challenging for effective disease control during this pandemic. Major variants identified to date include B.1.1.7, B.1.351; P.1; B.1.617.2; B.1.427; P.2; P.3; B.1.525; and C.37. Globally, large unvaccinated populations increase the risk of more and more variants arising. With successive waves of COVID-19 emerging, strategies that mitigate against community transmission need to be implemented, including increased vaccination coverage. For treatment, convalescent plasma therapy, successfully deployed during recent Ebola outbreaks and for H1N1 influenza, can increase survival rates and improve host responses to viral challenge. Convalescent plasma is rich with cytokines (IL-1β, IL-2, IL-6, IL-17, and IL-8), CCL2, and TNFα, neutralizing antibodies, and clotting factors essential for the management of SARS-CoV-2 infection. Clinical trials can inform and guide treatment policy, leading to mainstream adoption of convalescent therapy. This review examines the limited number of clinical trials published, to date that have deployed this therapy and explores clinical trials in progress for the treatment of COVID-19.

## 1. Introduction

Coronaviruses are a broad and diverse group of ssRNA (+) viruses that cause a range of infections across many species. The most notable infections affecting humans include: the common cold, Severe Acute Respiratory Syndrome (SARS), Middle East Respiratory Syndrome (MERS), and COVID-19 [1]. The first human coronavirus (B814) was identified from human adult respiratory tract in embryonic tracheal organ cell culture [2,3]; when intranasally innoculated, the virus caused a respiratory infection (common cold) in a large proportion of human subjects [3]. A further virus with the same “unusual features”, 229E, was cultured human tissue [4]; both viruses were ether-sensitive (likely having a lipid-coat) and unrelated to paramyxoviruses or myxoviruses [3]. McIntosh et al. (2004) reported multiple strains of ether-sensitive viruses from human respiratory tract samples and called these viruses ‘OC’ [5]. Electron microscopy of fluids from B814-infected organ cultures examined by electron microscopy showed 80–150 nm membrane-coated, pleomorphic particles with widely spaced club-shaped surface projections [6]; these were also observed for 229E and OC viruses. This new virus group was named coronavirus (‘corona’ due to the crown-like appearance of the surface projections) and it later became a new virus genus [7,8]. The genus comprises a range of human and zoonotic viruses, include: mouse hepatitis virus; infectious bronchitis virus; and transmissible gastroenteritis virus of swine. 

The first documented case of a human coronavirus exhibited symptoms of the common cold [2]. Patients presented with flu-like symptoms in 2001; 17 were shown to be infected with a coronavirus [9]. Most CoV infections were considered to result in mild infection including 229E and NL63 (belonging to group I coronaviruses that include NL and New Haven coronaviruses), but in the last decade, three coronaviruses—SARS-associated coronavirus (SARS-CoV); Middle East respiratory syndrome coronavirus (MERS-CoV), and SARS-CoV-2 emerged that resulted in severe morbidity and mortality in human populations [7,8].

The outbreak of severe acute respiratory syndrome (SARS), a contagious and potentially fatal disease, caused by SARS-CoV emerged in China in November 2002, rapidly spreading to 24 countries [10,11,12,13,14]. Between November 2002 and July 2003, 8098 confirmed cases of SARS and 774 deaths were reported worldwide with a fatality rate of 9.6% [14]. The SARS epidemic provided evidence that animal coronaviruses could jump species and cause significant human disease [1,15]. 

**Figure 1:**
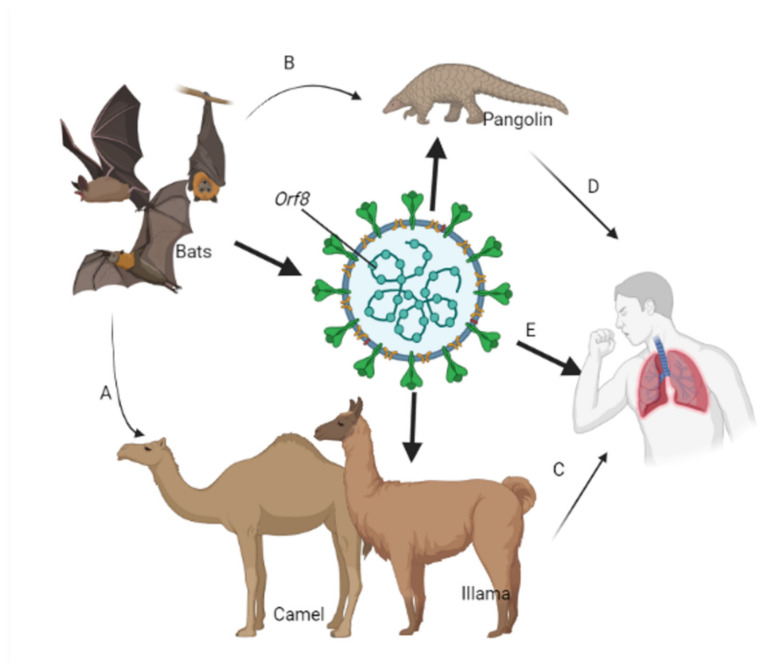
Zoonotic transmission of coronaviruses. Coronaviruses originally from wildlife species such as bats have undergone evolutionary changes to generate SARS-CoV and MERS-CoV genotypes through genetic recombination especially in the *Orf8* and S proteins. SARS-CoV-2 is transmitted to other wildlife species either through the domestic (**A**) or the sylvatic (**B**) cycle. Human interaction with host species through livestock communal activities (**C**) and wildlife poaching (**D**) leads to the introduction of SARS-CoV-2 variants in susceptible populations. Infections in humans are complicated by mutations in the SARS-CoV-2 genome (**E**), making routine control of infection challenging.

In 2012, another new viral respiratory illness emerged in Saudi Arabia; Middle East Respiratory Syndrome (MERS) MERS-CoV emerged with a fatality rate of up to 35%. MERS-CoV infections are transmitted from human to human, but dromedary camels are a major zoonotic reservoir host for the virus [16] (Figure 1). 

In 2019, another novel viral human respiratory pneumonia emerged in Wuhan, China called (COVID-19) caused by a new virus severe acute respiratory syndrome coronavirus 2 (SARS-CoV-2) [15,17]. Following the first outbreak of the SARS-CoV-2 in Wuhan, currently (as of 21 June 21, 2021), the global statistics of COVID-19 are as follows: total countries affected = 222; total cases = 179,369,956; total deaths = 3,884,375, new cases = +124,038; total recovered = 163,954,265; active cases = 11,531,316 [18].

### 1.1. Origin and Spread of SARS-CoV2

SARS-CoV-2 and SARS-CoV both likely originated in bats [19]. SARS-CoV-2 is believed to have jumped to humans from an intermediate and as yet undetermined species, sold at a “wet market” (the Huanan Seafood Wholesale Market) in Wuhan, China (Figure 1) [20]. China informed the World Health Organization (WHO), late in 2019, of a cluster of new pneumonia cases with unknown etiology, and by 3 January 2020, 44 cases had been reported. In early 2020, 2019-nCoV spread rapidly through the Asia-Pacific region and globally. The Thai Ministry of Public Health reported its first case of COVID-19 (imported from Wuhan, China) on 3 January. Japan reported its first case imported from China on 15 January. On 20 January, the Southern region of Korea, through its International Health Regulations (IHR) Focal point, reported its first case. On 23 January, the United States of America registered its first case. On 24 January, Vietnam reported its first case that was not related to travel from China. On 24, 25, and 26 January, the governments of Singapore, Australia, and Malaysia, respectively, confirmed their first cases. By 27 January, cases were reported in Canada. By 28 January, Cambodia, Germany, and Sri Lanka had all reported cases. On 30 January, cases were reported in the Philippines and India, and Japan reported cases on 6 February. Infections reached Europe in late January 2020; the first case was reported in Italy on 31 January; the Russian Federation on 1 February; UK, Belgium, and Sweden reported first cases on 5 February [21].

### 1.2. SARS-CoV-2 Infection

SARS-CoV-2 is transmitted by human-to-human contact, spreading through airborne droplets [22,23], or by surface contact. Individuals can be asymptomatic for the virus or symptomatic showing a variety of mild to severe respiratory and non-respiratory symptoms, including difficulty breathing, fever, headache, tiredness, persistent cough, myalgia, anosmia, and ageusia [24]. The virus infects the epithelial cells of the respiratory system damaging the cilia and leading to the secretion of inflammatory mediators that cause local inflammation and swelling and increased nasal secretions [25,26]. These reactions lead to airway obstruction and irritation of the mucosa lining in the upper respiratory tract. Uncommon features included diarrhea and dyspnea. SARS-CoV-2 has been detected in blood and isolated from bronchoalveolar lavage fluid samples. The virus has not been observed in the feces and urine of COVID-19 patients [27,28].

Genome analysis of SARS-CoV-2 shows two major lineages (L and S), which is well defined by two different single-nucleotide polymorphisms. The more recent ‘L’ lineage is more aggressive, spreads faster, and is more prevalent than the ‘S’ lineage [29].

### 1.3. Molecular and Immunopathogenesis of SARS-CoV-2 Infection

The clinical course of COVID-19 following SARS-CoV-2 infection has three phases [30,31].

Stage 1: Asymptomatic stage (1–2 days of infection). Following inhalation of SARS-CoV-2, the virus binds to the epithelial cells in the nasal cavity, where it replicates [31]. Angiotensin-converting enzyme 2 receptors (ACE.2) are the main receptors for SARS-CoV-2, and viral propagation occurs with minimal innate immune response. The viral load is low, but the person is infectious. The virus can be detected from nasal swabs using diagnostic polymerase chain reaction (PCR) tests and the viral load quantified using Real-Time PCR [23,24,32,33].

Stage 2: Upper airway and conducting airway response. The virus migrates down the respiratory tract, inducing a more vigorous innate immune response. The virus is detected from nasal swabs or from sputum. There is now a clinical manifestation of illness. Innate response cytokine levels (including for CXCL 10) are high and are determinants for disease prognosis. Infected cells produce β- and λ-interferons, with CXCL 10 being the major interferon response gene, which is an essential marker for COVID-19 [34,35]. Around 80% of infected individuals suffer only mild illness localized in the upper respiratory system and conducting zone, which can be managed by conservative supportive care and with at-home monitoring [23,32,33].

**Figure 2:**
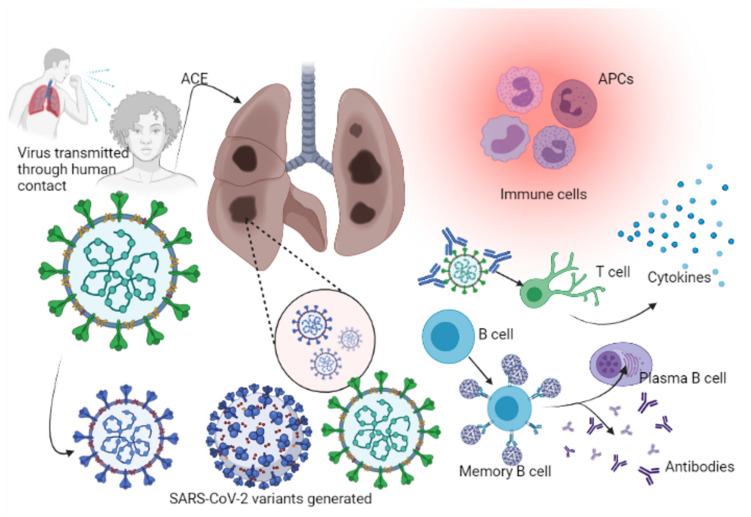
Immunopathogenesis of SARS-CoV-2 in humans. Colonization by SARS-CoV-2 is enhanced by ACE through which further viral replication takes place. Increased viral exposure leads to the generation of variants. Immune cells (APCs) including basophils, neutrophils, macrophages, and monocytes help to identify the infection. APCs work with T cells by binding to specific T cell receptors, which leads to the activation of CD4 and CD8 cells and the production of cytokines. The humoral response involves B cells, which are activated once an APC cell presents the antigen through the B cell receptors, leading to the activation of memory B cells and plasma B cells for the production of more antibodies to neutralize infection.

Stage 3: Hypoxia, ground-glass infiltrates, and progression to acute respiratory distress syndrome (ARDS). In 20% of cases, COVID-19 infection will progress to stage 3, which is associated with pulmonary infiltration; prognosis is uncertain and patients suffer high mortality [36]. The virus reaches the lungs and infects pneumocytes, mainly the type II alveolar cells. As with SARS-CoV and influenza viruses, SARS-CoV-2 preferentially infects subpleural and peripheral type II pneumocytes [32]. Propagation within type II cells leads to the release of a large number of viral particles that induce apoptotic cell death of the alveolar cells, resulting in the emergence of a self-replicating pulmonary toxin following the spread of infection to type II cells in adjacent units. Progressive death of type II cells in numerous lung areas triggers epithelial regeneration via secondary mechanisms. By infecting type II alveolar cells, the virus indirectly affects the type I cells (since type II cells are the precursors of type I cells and type II pneumocytes repair the alveolar epithelium when type I cells are damaged). Infection results in diffuse alveolar damage with fibrin-rich hyalin membrane and scanty multinucleated giant cells. The regeneration process leads to severe fibrosis and scarring [37], as a result of immuno-inflammatory processes [37]. Recovery depends on a strong innate and acquired immune responses and robust epithelial regeneration [38] (Figure 2). Immunocompromised individuals, including the elderly and those with chronic illnesses (cancer, diabetes mellitus, and chronic lung disease), are at increased risk for severe illness. The elderly and those with chronic lung disease have reduced epithelium regenerative ability and decreased mucociliary clearance, which enable viral spread within the alveolar cells [23,33].

### 1.4. SARS-CoV-2 Variants

All viruses, including the causative agent of COVID-19 (SARS-CoV-2), evolve, and these changes can significantly affect viral properties including transmission, disease severity and impact on diagnosis, and response to vaccination. Since January 2020, the WHO, researchers, national authorities, and expert networks within institutions have been monitoring the evolution of SARS-CoV-2 [39]. By late 2020, SARS-Cov-2 variants were emerging that were considered to pose a risk to global public health. Variants fall into two categories, Variants of Concern (VOCs) and Variants of Interest (VOIs), and they are characterized within each group using letters of the Greek alphabet (Delta, Gamma, Beta, and Alpha) [39,40,41].

A Variant of Interest (VOI) is a variant that in comparison to the reference virus has mutated, giving rise to new phenotype(s) and leading to multiple community transmissions/clusters/cases, as has been observed emerging in multiple countries. A Variant of Concern (VOC) will meet the definition of a VOI, but it has also been shown to be associated with one or more of the following: an increase in transmissibility or shows a detrimental change for COVID-19 epidemiology; an increase in virulence or change in clinical disease presentation; or will result in a decrease in the effectiveness of public health and social measures or available diagnostics, vaccines, and therapeutics; see Table 1 [39].

Mutations in SARS-CoV-2 that generate variants that may reduce the effectiveness of vaccines is a major public health concern. Mutations that reduce vaccine effectiveness will impede the development of ‘herd immunity’ and undermine local and global vaccination strategies [42]. The first variant of SARS-CoV-2, 501Y.V1 was observed in Europe [40] that was shown to influence host antibodies [43]. Mutations in RNA viruses can arise as ‘drop-off mistakes’ during viral replication [44,45,46,47,48], from gene diversity by recombination [48] and by RNA-editing mechanisms as part of natural host immunity [49]. The D614G mutation is associated with changes in the S protein of the SARS-CoV-2 genome, making it qualify as and classified as a VOC called B.1.1.7 [50], and D614G is associated with high viral load [51]. No correlation has been shown between the B.1.351 SARS-CoV-2 variant in Europe with disease severity. However, the B.1.351 variant has proved problematic for most NTB mAbs, with antagonist linkage to convalescent plasma increasing to around 30-fold [52]. SARS-CoV-2 variants are believed to be responsible for viral re-emergence in Manaus, Brazil i.e., B.1.1.28 (93, 37%), P.1 (24%), B.1.195 (20%), B.1.1.33 (12%), and P.2 (9, 4%). P.1 was identified as a VOC by January 2021 [53].

Surprisingly, SARS-CoV-2 has shown minimal diversity since its adaption in humans. Skewed mutant-bias from the SARS-CoV-2 gene delineation has been reported [54,55,56,57], and 98% of viral mutants had compromised K-mers for homoplastic sites. Domingo-Calap et al. observed C-C-A enrichment in dedicated editable APOBEE-RNA enzymes [54]. Many branches in the phylogenetic have a low genetic-defection range with dominant stray heads in familial alternate alleles in progeny. The variability in viral transmissibility of various viral strains can be estimated by analyzing the percentage of wild-type genome. Identification of the E484 spike protein involved in covalent association with serum molecules has 10-fold diminished apolar field restrictions, which is an observation common to the B.1.351 and P.1 types [58,59]. There are taxonomic efforts to classify viral sub-lineages [43]; however, inconsistencies exist due to restrictive health policies in some countries. Great caution should be exercised in declaring whether specific mutations contribute to changes to viral phenotype, allele pairing, etio-demographic factors, or epidemiological process; compared to SARS-CoV-2/D614G isolated in hosts by the so-called recurring selection.

### 1.5. Disease Severity Classification

COVID-19 patients are considered critical if they: show (a) severed respiratory distress necessitating artificial respiration (b) shock, requiring vasopressor management and associated with raised lactate concentration greater than 2 mmol/L even with sufficient replacement of fluids, or (c) show indication of multiple organ failure and therefore require the patient to be admitted to the intensive therapy unit (ITU) [60]. Severe diseases in COVID-19 cases are seen in around 20% of cases [60], and severe cases of COVID-19 are associated with high death rates (over 50% of cases). COVID-19 can progress rapidly to severe with an average of 9 days from the time of onset of symptoms to acute respiratory distress syndrome [17]. Early identification of infection can prevent progression to severe COVID-19 infection [61].

### 1.6. Clinical Features

#### 1.6.1. Signs and Symptoms

Around 17% of COVID-19 patients will develop acquired acute respiratory distress syndrome, which is the main fatal complication amongst these patients [61]. Fever is common in COVID-19 infection and is not an indicator for progression to severe disease. Other clinical characteristics for severe COVID-19 include myocardial damage and chest pain from hypoxemia or virulence of the virus. Dyspnoea and chest pains have been shown to occur more frequently in patients with severe COVID-19, and focusing on the occurrence of these rarer symptoms of COVID-19 during early stages of the disease, especially amongst the elderly who are at a higher risk for severe COVID-19 [61,62], can be predictive of risk of severe disease. Disease severity has also been correlated with lymphopenia. Lymphopenia is rarely found in SARS-CoV2 infected children, which is one of the main reasons contributing to the low mortality rate of children from COVID-19. The rate of severe disease increased with age; occurrence of severe COVID-19 is not only due to the weakened immune function of the elderly population but also to variable susceptibility to the virus in different age groups [63].

High levels of fatal thromboembolic events in COVID-19 disease have been observed [64,65]. Autopsies have shown 58% deep vein thrombosis in COVID-19 patients and the occurrence of pulmonary embolism in 33% of cases [66,67]. High incidence of arterial thrombosis including stroke and acute coronary syndromes has also been documented [68]. The D-dimer is a useful biomarker for disease severity [69] and is used to predict disease outcomes [70]. The application of anticoagulant thromboprophylaxis in the management of venous thromboembolism (VTE) cases that occur often is recommended [29].

#### 1.6.2. From Bedside to Bench-Side

##### C-Reactive Protein (CRP) Levels Evaluation

CRP is a valuable marker for systemic inflammation suggestive of infection or tissue damage. CRP levels correspond to COVID-19 severity arising from SARS-CoV-2 infection and are correlated with the development of acute lung damage [27,71]. Some studies have shown no notable changes in the level of liver enzymes in patients with severe COVID-19 [71,72], but chest CT scans indicated that most COVID-19 patients show significant pathological changes in lung tissues, which is suggestive of SARS-CoV-2 infection being related to lower respiratory tract pathology. The degree of pulmonary damage is correlated to disease severity and its prognosis. COVID-19 patients with multiple lesions in both lungs are more likely to be severe cases; visible changes in the lungs on chest CT scans are more notable in patients with severe disease [73].

##### SARS-CoV2 Antibody Detection: ELISA

Profiling early humoral responses to diagnose and assess the prognosis of COVID-19 disease uses immuno-enzyme testing techniques to detect plasma/serum antibodies (Abs) against SARS-COV-2 in patients before CPT administration (−1 day), and on days 1, 3, 7, and 12. Combining IgM and IgA makes the assay more sensitive, and IgG, IgM, and IgA analysis can be performed at once [74,75]. ELISA specificity for SARS-CoV-2 IgM and IgG were 96% (144/150) and 100% (150/150), respectively, and the sensitivity of the test was 100% (150/150) for IgM and 99.3% (149/150) for IgG. SARS-CoV-2-SP-IgM and SP-IgG antibodies were detectable on day 1 following hospitalization in 12.5% of cases, and SP-IgM started to decrease/reached its peak at around 22–28 days, becoming negative after 3 months in 30% of cases and negative at month 7 in 79% of these patients after onset; IgG reached its peak around day 22–28 and stayed at a high level for the longest time (4 months) before dropping abruptly at 7 months [76].

##### SARS-CoV2 Neutralization Abs in Convalescent Plasma Therapy

Convalescent plasma (CP) therapy is a treatment for COVID-19. Information as to the amount of neutralizing antibody (NAb) present in donor blood plasma and its importance in COVID-19 patients’ treatments is largely unknown. NAb titers in the CP of the donor and patients of COVID-19 should be evaluated before, during, and after transfusion with CP [77] to determine the effectiveness of CPT during the management of severe COVID-19 cases (measuring the level of neutralizing antibodies by titration method) [74]. Samples are analysed for cytopathological features, samples with the highest dilution factor that inhibits SARS-COV-2 activity are those with the highest concentration of the neutralizing antibodies. A clinical trial involving 10 severe adult cases of COVID-19 first confirmed by molecular techniques suggested that a single dose of CP (200 mL) with supportive management and antiviral drugs was safe. Nabs reached high levels in COVID-19 patients within 3 days, leading to improvement of the clinical presentation of patients, disappearance of the SARS-COV-2 viruses, and improvement in pulmonary radiological findings within 7 days of treatment [73]. The test also measures the neutralizing antibody titers of CP derived from recovered donors before it is transfused to the patient [74,75].

On 24 March 2020, the Food and Drug Administration, USA (FDA) issued guidelines for CP donation and application of CP in COVID-19 patients under the emergency investigational new drug (IND) applications, and the IND regulatory pathway (21 CFR 312). The guidelines proposed initial evaluation of the NAb if the assay is accessible, followed by the employment of donors with NAb titers of ≥1:320 (FDA, 2020) later reviewed to NAb titers ≥1:160, or 1:80 in absence of a unit with a titer of 1:160 [78]. SARS-CoV-2 NAb assays are not commonly available in most countries [79], and CP therapy has mostly been undertaken without Nab assays in CP before transfusion [80].

## 2. Management and Prevention of COVID-19

### 2.1. Control of the Spread of COVID-19

Guidelines for the control of transmission of the coronavirus by the WHO and the European Centre for Disease Prevention and Control (ECDC) focus on the protection of infected patients by health professional workers. The WHO published universal guidelines promoting the isolation of infected individuals from their consistent immediate neighbors and the implementation of precautions for direct contact and aerosol infection. ECDC published guidelines recommending contact avoidance of sick people, especially those with a cough, avoiding crowded areas and places where live or dead animals are handled, frequent hand washing with soap and water, and use of alcohol-based disinfectant before eating, after visiting the toilet, and after any contact with animals [81,82]. Preventive measures have become more critical with significantly constrained supplies of the COVID-19 vaccines [83].

### 2.2. Treatment of SARS-CoV-2 Infection

Treatment of COVID-19 requires supportive therapies including the use of analgesics, antipyretics and antibiotics, hydration, and artificial respiratory support. Therapeutic options including antimalarials, antivirals, and vaccines are under study. While there are claims that ribavirin and interferon-alpha offer synergetic effects in the early stage, others suggest mycophenolic acid as monotherapy. Although there are appreciable advancements in the discovery of therapeutic agents, the results are unsatisfactory, requiring further clinical research [81,82]. Newer strategies for treating COVID-19 patients involve repurposed drugs and the application of targeted novel formulations to SARS-CoV-2.

Convalescent therapy is a therapeutic option that can be administered for severe cases of COVID-19 involving the administration of blood plasma from previously ill but recovered COVID-19 individuals (donors) to COVID-19 patients (recipients) [84]. Emil Behring was first to demonstrate taking plasma from individuals recovered from diphtheria and using it to treat newly infected diphtheria patients [85]. Knowledge remains scares as regards immunological responses and antibody production following SARS-CoV-2 infection. Antibodies are highly specific, binding to the invading organism and targeting the destruction of the infectious agents through the major histocompatibility complex (MHC) via human leukocyte antigens (HLAs), which is a complex coordinated mechanism involving antigen-presenting cells (APCs) [86]. Pathogens also activate B lymphocytes which ultimately differentiate into memory cells. Re-exposure to these pathogens causes B-memory cells to produce immunoglobulin G (IgG) antibodies, generating a secondary immunological response that is usually faster and stronger. Introducing live or attenuated pathogens into the body to provoke antibody generation is the basis of viral vaccines [86,87].

## 3. Convalescent Plasma Therapy (CPT)

CPT was used in the clinical management of Ebola cases around the world following Ebola virus (EBOV) outbreak in West Africa in 2014. CPT has been assessed in the USA and in West Africa, but clinical trials subsided with falling Ebola cases [88]. Observational studies undertaken during the first phase of SARS in 2002 showed improvement in patients treated with convalescent plasma therapy, but these were individual case studies [88]. No large-scale randomized trials for CPT have been performed to date. From the limited case studies reported to date (each study concluding on the safety of plasma therapy and improved patient outcome), the WHO have designed guidelines for CPT for Ebola infections. Limitations of the studies include small sample sizes (maximum of 10 patients) and a lack of control patients (not treated with CPT) [88]. There is a need to determine the effectiveness of CPT for COVID-19 disease deployed during the pandemic, and clinical trials are ongoing that will provide data to justify (or otherwise) the use of CPT for COVID-19 patients [89,90,91,92].

### 3.1. Negative and Positive Aspects of Convalescent Plasma Therapy

#### 3.1.1. Limitations and Negative Aspects of CPT

There are administrative, logistic, and ethical barriers, as well as negative aspects of the therapy itself for CPT. Patients are administered with approximately 500 mL plasma, after which their prognosis is carefully monitored. The condition of some patients may worsen due to antibody-dependent enhancement (ADE), in which antibodies specific for the disease-causing viruses bind to cells expressing receptor sites for those antibodies. The cells become susceptible to viral infection, increasing the viral replication rate. This occurs in dengue infection and is of concern in MERS coronavirus infection. A second challenge is the possibility of the preformed antibodies to inhibit the immune response of the host from responding adequately [93]. This can occur from antibodies passed on from mother to offspring, which can prevent infants from responding adequately to vaccination and is the reason vaccines administered earliest started at eight weeks postnatal [90,91,92].

Administrative and logistical barriers such as identification, consenting, collecting, and testing of donors is problematic, as is access to suitable assay equipment, etc. to process CP. CPT use in developing countries is constrained mainly due to systemic and transfusion-specific challenges including inadequate capacity for donor mobilization and collections lack functional health care systems, among others [94,95].

Donor eligibility requires donors consenting to donate plasma should meet the eligibility criteria for standard blood donation; e.g., for CPT, the donor must be negative for the SARS-CoV-2 test, free from COVID-19 symptoms, etc. [16,96,97]. A lack of neutralizing antibodies in the donor (recently recovered patient) plasma can hinder the preparation of CP for treating cases, and these antibodies last for only a few weeks to months. CP treatment also requires large infusion volumes (200–2400 mL) [98], and there is no available standardized transfusion dose of CP, i.e., the dose depends on the patient. The time of administration also determines the outcome; i.e., the best outcome occurs in those recipients/patients who have received CP transfusion before the development of the humoral immune response [99].

There will be a large imbalance between the number of recovered and active/positive cases, and it is a challenge to meet the demand for a large amount of plasma needed to treat the large number of patients being infected [97]. Mutations are common among viruses, including the coronaviruses, and there is a risk of diminishing antibodies.

Standard operating procedures for using convalescent whole blood and convalescent plasma among people with different diseases do not exist. Although studies to date support the safety and the efficacy of CPT to treat infectious diseases such as COVID-19 [92,93,94], uncertainty exists regarding the safety and efficacy of CPT therapy for people with infectious diseases. Although there is a theoretical reason to think that antibodies in the blood or plasma of individuals who have survived viral diseases can reduce the viral levels of severely ill patients, research regarding this is still ongoing [17,96].

CP transfusion may induce transfusion-associated reactions ranging from mild to adverse reactions; these reactions include fever, allergy, life-threatening bronchospasm, acute lung injury, and aged persons and patients with renal and cardiorespiratory disorders may get circulatory overload [100]. Other rare adverse reactions associated with CPT include phlebitis, generalized jaundice, evanescent facial red spot, and ADE transfusion-associated infections such as SARS-CoV-2 itself, *Treponema pallidum* and hepatitis B are rare with CPT but can occur. CP administration carries the risk of re-infection, since the process involves infusion of passive antibodies that may weaken the recipient’s immune system, causing inhibition in the production of pathogen-specific antibodies and resulting in re-infection by the pathogens of the disease that is being treated [97].

Monoclonal antibody cocktails and immunoglobulin preparations could be effective preventive and treatment options to resolve efficacy issues; however, if standard precautions and blood safety strategies are followed, CPT is expected to be safe [17,96].

#### 3.1.2. Positive Aspects of CP Therapy

There is a positive risk–benefit balance with CPT and people with infectious diseases. CP transfusion is well tolerated by most patients, and improved clinical outcomes in severe cases of patients with COVID-19 are observed, although some adverse effects are also seen [98]. CPT has been used successfully for treating and reducing the mortality of people infected with South American hemorrhagic fevers, Ebola, SARS, MERS, Hantavirus infection, and COVID-19 [92,93,97].

Regarding clinical efficiency of CP therapy, preliminary studies show promise. A study on five critically ill COVID-19 patients with acute respiratory failure and artificial ventilation found that all showed improvement after the administration of CP containing SARS-CoV-2 antibodies, combined with lopinavir/ritonavir and interferon treatment between days 10 and 22. Patients showed an incremental increase in antibody levels, decrease in viral load, and alleviation of symptoms of COVID-19, including ARDS [97]. CPT can significantly reduce the case fatality rate for viral infections compared to supportive and other treatments [91,96]. Very low mortality levels are associated with CPT; patients receiving CPT, especially in the early phases of disease onset, show significantly very low mortality rates. The mortality rate in patients receiving CPT before 14 days of onset was found to be 6.3% and after 14 days was 21.9% in patients infected with SARS-CoV-1; no mortality was reported in patients receiving CPT for patients suffering from SARS-CoV-2 in five other studies [101].

CPT also provides beneficial effects attributed to plasma components such as organic compounds, water, proteins (albumin, globulins, coagulation, and antithrombotic factors complement components), and inorganic slats [102]. The beneficial effects of CPT include replenishment of coagulation factors vital in hemorrhagic fevers e.g., Ebola, immunomodulation role due to anti-inflammatory cytokines and antibodies, and the maintenance of colloidal osmotic pressure body fluid compartments mainly by albumin [97,103]. CPT has proven its ability to reduce viral load in influenza patients [104], and a meta-analysis demonstrated a significant reduction in mortality among patients treated with convalescent plasma or serum in severe viral acute respiratory infections [105].

### 3.2. Convalescent Plasma (CP) for Treatment of Infectious Diseases: PAST Experiences

The emergence of newer antimicrobial agents (antivirals, antibiotics, and vaccines) led to a reduction in the use of convalescent serum or plasma therapy, but with the emergence of novel infections with no specific drugs, there has been renewed interest in immune therapies derived from immune survivors [106,107].

### 3.3. Convalescent Plasma Therapy for Ebola

#### 3.3.1. Plasma Transfusion and Convalescent Whole Blood for Treatment of EBOV

Ebola virus causes a viral hemorrhagic fever called Ebola/Ebola Virus Disease (EVD). This enveloped negative-stranded RNA virus was first identified in 1976 in southern Sudan and later in northern Zaire. EBOV has traversed equatorial Africa for decades, with outbreaks occurring more often since 2000 [108]. EVD is a zoonotic infection with natural/intermediate hosts (rodents, fruit bats, chimpanzees, or monkeys), and the virus is transmitted from wildlife to people through fluids and also seen in human-to-human transfer from infected to uninfected individuals. The clinical presentation of EVD begins with non-specific symptoms including chills, fever, myalgia, and malaise, advancing into a severe hepatitis/gastroenteritis phase accompanied by nausea, vomiting, diarrhea, and anorexia, leading to hypovolemia, electrolyte imbalance, and metabolic acidosis [108]. EVD can cause multiple organ failure and has a 21.2% to 60.8% case fatality rate [109].

EVD management is largely supportive with fluid and electrolyte replacement and symptomatic treatment. Clinical trials have explored treatment options [110,111,112,113,114].

CPT was used for the management of EVD during the Zaire Ebola epidemic of 1976; here, one recipient fully recovered after being transfused with two units of CP (200–300 mL per unit) [115,116]. During this outbreak, 26 convalescent donors donated 201 plasma units, and samples with EBOV antibody titers < 1:64 were selected for use. Even though CP was found to contain microfilaria, this did not affect processing [117]. This plasma was also stored for subsequent emergencies [102].

Convalescent whole blood administration was deployed during the Rvsv-Zaire Ebola virus (ZEBOV) epidemic in Kikwit, Democratic Republic of Congo, in 1995 [118]. Eight patients received transfused EBOV convalescent whole blood, seven of whom survived (12.5% case death rate for an infection that is considered 80% fatal). An experiment conducted in primates illustrated the relevance of passive immunity conferred by the convalescent transfusion of whole blood [119], and a study in mice showed the relevance of passive polyclonal immune serum transfusion. Mice with a serious deficiency in the immune system showed a survival rate of 100% in mice challenged with a lethal dose of ZEBOV, but the mechanism of protection could not be established [13]. Recently, similar experiments in primates yielded similar results [120].

ZMapp, a drug made up of individual monoclonal antibodies against EBOV was the first experimental agent for EVD in Europe and USA but had limited supply [82]. ZMapp is a monoclonal antibody cocktail (mAbs) made against EBOV. The experimental drug is made of 3 mAbs of IgG class, which bind to three different epitopes of the EBOV surface glycoprotein [121]. Abnormal humoral responses were noted after ZMapp use in patients with EVD in the US, and other forms of immune therapy were used to manage the remaining EVD patients [121]. The first trial of convalescent plasma in the management of EVD cases in the US and West Africa involved transfusing plasma from a survivor to an EVD patient at University of Nebraska Medical Center (UNMC) [122], and the practice of using ECP with EVD patients has been adopted since then in the US and Europe. The WHO recommended the use of CP in EVD patients as a treatment and provided national health authorities and transfusion organizations with guidelines regarding the collection and processing of convalescent whole blood or CP from EVD-recovered patients [123]. A health worker (51 years of age) who was infected with EBOV and received two doses of CP on the 9th and 10th days of sickness showed complete recovery and absence of the virus in their plasma (negative EBOV RNA test) within 28 days. Another severe EVD case in Sierra Leone received six doses of CP on separate days following sickness. He also received TKM-100802 in the initial phases of the illness (discontinued due to severe organ damage from it), and although the patient was in a critical state (on supportive respiration and dialysis), he recovered fully and tested negative for EBOV RNA within 44 days. Although the recovery of the above two EVD patients was attributed to CP and TKM-100802, the use of the agents was uncontrolled; therefore, one cannot dispute the fact that the recovery of the patients could be attributed to other factors such as adequate supportive care [123].

In Europe, CP therapy was used with caution for EBOV, following the development of transfusion-related acute respiratory distress following treatment in an EBOV-infected nurse [124]. It was suspected that the most common cause of the distress was linked to CP transfusion, as they found no human leukocyte or neutrophil antibodies in the donor and therefore no other reasonable cause of hypoxia and associated lung damage [123]. Further studies are recommended to investigate the most likely complications related to EVD and CP transfusion, such as pulmonary complications.

#### 3.3.2. Current Research Concerning the Use of Ebola Convalescent Plasma

There are two trials in the USA gathering data and on CP for EBOV. Phase 1 (open-label safety study) at the Emory University and UNMC is examining the effect of passive immune therapy on acute EBOV infection CP from volunteer participants that had recently recovered from EVD [125]. The aim is to collect and process plasma from EVD survivors and store it to form an inventory. Plasma was collected using apheresis, and pathogen inactivation was performed. NIH are gathering blood from individuals who were previously exposed to or vaccinated previously to Ebola, with a broader view of creating an infection recovered CP inventory [122,126].

A number of clinical trials have been undertaken to determine the efficiency and safety of CP for the EBOV treatment in West Africa and the United States [127,128]. In a study in Liberia, Sierra Leone, CP was transfused from two BVD recovered donors; the primary outcome was based on the viral load of EBOV in the recipient’s blood [129,130]. A total of 200 EVD patients were enrolled and received ECP from two different donors each, the outcome being dependent on improvement in survival rate by the 14th day [129].

Another CP study on EVD is being conducted in Sierra Leone by the University of Liverpool with ETU in Freetown targeting the disease in its initial stages. They plan to involve 200 EVD patients to be transfused with CP from a single individual and 100 control patients. The outcome will be measured according to all-cause mortality on day 14 following administration, and the data are not yet available [131,132].

#### 3.3.3. Convalescent Plasma Therapy for Patients with MERS-CoV

Treatment with CP is currently being recommended as a possible form of treatment in the management of MERS. Clinical trials for CP have been embarked on, such as those supported by King Abdullah International Medical Research Centre in Saudi Arabia [124].

The first case of MERS-CoV infection was reported in Saudi Arabia in September 2012 [133]. By 30 September 2015, there were 1589 confirmed cases of MERS-CoV infection, with 567 associated deaths [134]. The International Severe Acute Respiratory and Emerging Infection Consortium (ISARIC) and Public Health England put forward a decision support tool for clinical officers handling cases of MERS-CoV infection, where testing CP or other neutralizing antibody therapeutic methods (such as hyperimmune immunoglobulin) were recommended for the management of MERS-CoV infection [135]. For MERS, mortality as a clinical end point is difficult, considering the need for a large sample size, to achieve significant statistical power [136], and it might be useful to first determine the viral load for each patient, the donor’s plasma levels of neutralizing antibodies, and antiviral effects in a small open-label study before treatment. Such information would be useful in determining the design of the study and the most effective dose range of neutralizing antibody, informing doses for making anti-MERS-CoV antibody preparations for preclinical development. A two-phase study is recommended, where the first phase involves a collection of serum from donors with significant MERS-CoV antibody titers, while the second phase involves the treatment of MERS-CoV patients, evaluation of safety, and efficacy of CP infusion for subsequent adoption [137].

#### 3.3.4. Convalescent Plasma Therapy for Patients with Influenza A (H1N1) Infection

During the influenza A (H1N1) outbreak in Hong Kong, a randomized double-blind controlled study was carried out among critically ill patients to evaluate the effects of hyperimmune intravenous immunoglobulin. The Hong Kong Red Cross Blood Transfusion Service (BTS) had the responsibility of blood collection and preparation of CP following blood donation standards. In total, 1309 individuals were enrolled (had recovered from influenza A), of which only 493 were considered eligible for CP therapy donation. Only 301 individuals presented for apheresis plasma donation. At the end of the collection process, about 276 L of CP were fractionated to hyperimmune intravenous immunoglobulin (H-IVIG) [138]. Due to a large volume of plasma fractionated, some of the CP was used to treat some of the critically ill patients [138]. Treatment with CP or H-IVIG was linked with a drastic drop in viral load and mortality rate [22,139].

#### 3.3.5. Convalescent Plasma Therapy for Patients with SARS-CoV Infection

CP was employed for SARS during severe cases when the prognosis is poor despite being on supportive management [19,21]. However, it is imperative to consider the administration of CP in the early stages of SARS infection to improve efficacy, as suggested in a recent review (102), in which a significant decrease in the mortality rate following treatment with CP compared to patients on supporting care only without CP therapy [132]. Studies have determined that early administration of CP in the early stages of the illness provides the best clinical outcomes [134]. A previous study in 2003 evaluated the efficacy of CP therapy in managing 80 patients with SARS in Hong Kong [119]. Good outcome and discharge from hospital by day 22 following onset were observed among most of the patients who were administered with CP before day 14 of illness (58.3%) compared to 15.6% who improved without receiving CP. Additionally, CP therapy cured 66.7% of those who were PCR positive and seronegative for coronavirus at the time of plasma infusion compared to 20% cured without CP therapy [119].

### 3.4. Convalescent Plasma Therapy in the Management of COVID-19

There is presently no single drug treatment for SARS-CoV-2. Several clinical trials are ongoing to improve the outcome of the disease related to SARS-CoV2 infection. Drugs including lopinavir–ritonavir have been found to improve treatments in COVID-19 patients; however, more studies remain to be conducted [140]. Numerous vaccines are now being administered globally under emergency use including RNA vaccines (i.e., Pfizer/BioNTech and Moderna) and adenovirus vector vaccines (i.e., Oxford-AstraZeneca, Sputnik V, Janssen, Convidecia and Sputnik Light), inactivated vaccines (i.e., Sinopharm, QazCovid-in, Minhai, COVIran Barakat, Covaxin, and CoviVac), and protein subunit vaccines (i.e., Soberana O2, MVC Covid-19 vaccine, Abdala, and Zifivax) as well as viral vector vaccines (i.e., Sputnik Light and Convidecia) [141,142].

CP therapy has shown promise, but there is a need for a randomized trial aimed at assessing safety and efficacy; these are difficult to execute during a pandemic crisis [143], which is when accessing control patients is most difficult [144].

After SARS-CoV-2 seroconversion, the development of SARS-CoV-2 antibodies from infection (or immunization) can be detected in blood samples, and in those infected, the increased levels of antibodies are accompanied by a decline in viral load [99,145]. For CPT, plasma administration prior to SARS-CoV-2 seroconversion (<5 days post-exposure) is critical to avoid disease progression from mild to severe. This could prevent clinical deterioration, shorten hospital stay, and ultimately improve the survival rate of COVID-19 patients. A 50–80 kg patient is to receive 2 units of 200–500 mL for up to 10 days intravenously, with adjustment of the volume for patients outside this range of weight, slow rate infusion that is closely monitored to recognize and manage acute transfusion-related side effects such as fluid overload, as well as post-transfusion monitoring for lung and other systemic inflammatory or other side effects. Following the confirmation of adequate tolerance, the patient may receive the next transfusion [113,132].

Tang et al. conducted a retrospective study and described four critically ill patients who had their clinical signs resolved following the intravenous administration of 200–2400 mL of CP within 11 and 18 hours, including a pregnant woman [139]. Tian et al. had a contrary report following the observed failure to recover, with loss of more than 80% of critically ill patients despite the administration of CP, with the consequent halt of the study, but they concluded that CP administration must always be done at the early stage of the infection [24]. On the 26th of March 2020, the use of CP was recommended in China by the China Food and Drug Administration.

## 4. COVID-19 Convalescent Plasma (CCP)

COVID-19 convalescent plasma therapy involves the collection of plasma from an individual who was once infected with COVID-19, who has recovered and developed some antibodies against the SARS-CoV-2 virus and transferred it to another individual with COVID-19 infection [146]. Plasma obtained from individuals who have recovered from COVID-19 contains antibodies that may be deployed in combating the SARS-CoV-2 virus; therefore, treatment of COVID-19 patients with plasma obtained from the recovered patients could cause a faster improvement of the disease due to curbing of the SARS-CoV-2 virus by antibodies from the donor plasma [147].

A study among 23 COVID-19 patients revealed a temporal pattern of antibody response in 108 serum samples from the 23 patients [148]. Ten days after the appearance of clinical COVID-19 symptoms, antibodies, nucleoproteins, and the viral spike receptor-binding domain (RBD) were reported in most patients. Individuals infected with SARS-CoV-2 have been reported to have presented a higher level of antibodies compared with the observation among SARS-CoV-1 infected individuals. Zhao et al. also reported similar findings [149]; however, whether the presentation of higher antibody levels is a response to a more severe disease condition or are an ADE trigger resulting in more severe disease condition remains unclear to date. Most patients infected with SARS-CoV-2 will already have an antibody response, which raises concerns as regards the rationale for CP infusion. Estimates on the number of people likely to be hospitalized as a result of COVID-19 infection keep increasing globally. Considering the failure of hyperimmune globulin in most randomized controlled clinical trials to improve clinical outcomes in influenza A [137,150] or respiratory syncytial virus [148] and given the non-availability of randomized clinical trials on CP for any other viral disease, it is not wrong to assume that 1–2 U of CP in COVID-19 will be clinical relevance.

The outbreak of COVID-19 represents a major challenge to the healthcare sector globally. In wealthy nations, patients infected with SARS-CoV-2 often receive multiple therapeutic interventions simultaneously including CP and other experimental therapies [150]. Randomized controlled clinical trials will help us understand the benefit, lack of benefit, comparative value, or risk associated with the use of CCP [151].

### 4.1. Source of COVID-19 Convalescent Plasma

Acquisition of CP is via apheresis, where whole blood is collected from patients who have recovered from a particular infection assuming the donor has developed antibodies against the causal agent of disease [152,153,154,155]. CP is used as an emergency intervention [120,155,156,157,158], and critical to success is early administration after symptoms onset [120,159]. Antibodies transfused into a COVID-19 patient are expected to have an antiviral effect, interrupting virus replication and lowering viral load before the patients can mount their own humoral immune responses [159,160,161].

In Henan province, the first people who recovered from COVID-19 reportedly donated blood in mid-February 2020 [162]; 200–400 mL of plasma sample was separated from the whole blood for each donor, followed by gold immunochromatography for confirmation of seropositivity for anti-SARS-CoV-2 (SARS-CoV-2 IgM and IgG tests). Prior to donation, donors were confirmed to be afebrile for at least three days with monitoring of the resolved respiratory symptoms and consecutive SARS-CoV-2 nucleic acid negative test outcomes for at least 3 weeks following disease onset [163].

Few studies have assessed the efficacy of CP, and randomized case-controlled clinical trials will be important to determine the safety and effectiveness of CP before routine clinical deployment for COVID-19 patients [164]. During apheresis, in addition to neutralizing antibodies (NAbs), other proteins such as clotting factors, anti-inflammatory cytokines, defensins, pentraxins, natural antibodies, and other undefined proteins are collected from donors [165]. CP transfusion to infected patients may provide other benefits such as immunomodulation by improvement of the severe inflammatory response [166]. The latter could be the case of COVID-19 associated with over-activation of the immune system, with consequent systemic hyper-inflammation/cytokine storm often driven by various interleukins (IL-1β, IL-2, IL-6, IL-17, and IL-8), CCL2, and TNFα. This systemic hyper-inflammation may be associated with pulmonary damage, with consequent pulmonary fibrosis and compromise of pulmonary functional and structural capacity [167,168].

During SARS-CoV, influenza A (H1N1), and MERS-CoV epidemics, no adverse events were reported to be linked with the use of CPT. For Ebola, CPT was associated with mild adverse reactions such as skin erythema, nausea, and fever [130]. For the present COVID-19 outbreak, reports have shown CPT is safe, and CPT has not been linked with major adverse events. CP in terms of tolerability and potential efficacy is a strong candidate for evaluation for treatment during the current global pandemic [159].

In Sierra Leone, a small nonrandomized study revealed significantly lowered fatality among patients treated with convalescent whole blood compared to their counterparts who received the standard Ebola treatment [169]. Two Ebola patients transferred to the US who were treated with a combination of convalescent serum and an experimental drug also survived [127]. Other evidence suggesting the use of CP in viral infection are reported previously [170] and [171] in H5N1 and H7N9 outbreaks, respectively.

### 4.2. Risk–Benefits of CPT

COVID-19 convalescent sera could be potentially used for prophylactic treatment as well as therapy. During prophylaxis, serum administration could prevent COVID-19 disease in populations that are vulnerable and those with high risk to exposure [172]. The potential for passive antibody administration is being used as current vaccine therapies in the fight against infant severe respiratory syncytial virus (RSV) disease in the children at high risk [173]. Serum therapy has proven to be more potent in disease prevention than the actual treatment of the ailment [174].

Risks associated with convalescent sera include those that are known to be affiliated to transfer blood and products, leading to possible immunological reactions [159], but modern transfusion and compatibility screening techniques have reduced this risk to almost zero [175]. However, antibody-dependent enhancement of infection (ADE) has been seen in a variety of diseases of viral origin and involves an amplification of disease reaction within disease antibodies. Various ADE modes of action have been identified in coronaviruses, and there are concerns that antibodies to strains of coronavirus may in some cases amplify a response in the recipient [176]. ADE risks are associated with SARS-CoV-2 B-cell vaccines for certain populations based on age, variabilities in antibody levels over time, cross-reactive antibodies, and pregnancy [176]. Existing literature from the use of convalescent antibodies as therapy in patients with SARS1 and MERS (120) suggests that CPT is safe. Nevertheless, caution and vigilance must be taken into consideration [177].

Tang et al. described four critically ill patients with COVID-19 whose clinical signs resolved following intravenous administration CP, including a pregnant woman [139]. However, Tian et al. observed the loss of more than 80% of critically ill patients despite the administration of CP, necessitating the cessation of the study, concluding that CP administration must be used in the early stage of COVID-19 infection [24]. On the 26th of March 2020, CPT was recommended for COVID-19 treatment in China by the China Food and Drug Administration.

A previous study showed that passive antibody administration pre-vaccination with respiratory syncytial virus moderated antibody but not cellular immunity [178]. Clinical studies can assess the body’s response to passive antibody administration before being administered with convalescent antibodies. Therefore, available data on convalescent serum use imply that the payback of its use in high-risk populations (the elderly and the vulnerable) with prior infection history is greater than the associated risk. However, this does not rule out the fact that risk–benefit assessment is undertaken in all cases where convalescent serum administration is measured [179].

**Figure 3:**
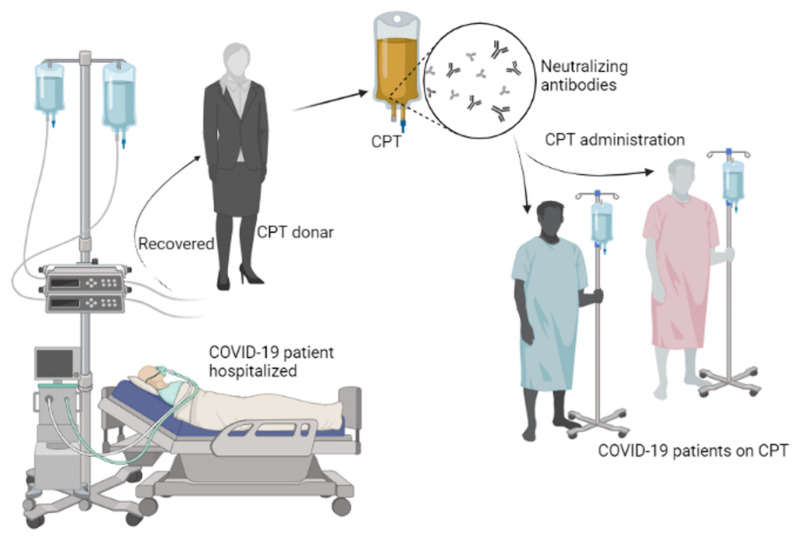
Adaptation of CPT for the management of SARS-CoV-2 patients. Immunity against SARS-CoV-2 is strongest amongst those who have had severe COVID-19. Recovered patients are ideal candidates as CPT donors. Active antibodies are administered to COVID-19 patients to help boost their immune system against infection.

For individuals recovering successfully from the COVID-19 virus with high neutralizing antibody titer values (Figure 3), serum can be extracted and used prophylactically to avoid contagion in highly vulnerable persons i.e., persons with underlying disorders, healthcare providers, and persons with contact to established cases of COVID-19. Convalescent serum should also be administered to infected persons to help build their immune systems and responses [161].

Eligible donors for COVID-19 convalescent plasma are individuals who have recovered from the virus at least for one to two weeks [179]. Overall health/history of past diseases, medical treatment, weight, age, risk behaviors, etc. criteria for regular blood donation all apply here. The donor should be screened in a laboratory for COVID-19 infection. Tests may be conducted by collecting nasopharyngeal swab specimen once or more or by collecting blood samples for molecular tests. Male donors are preferred; however, for the case of female donors, it is preferred to use non-pregnant females (161].

### 4.3. Convalescent Plasma and the European Blood Alliance (EBA)

The European Blood Alliance (EBA) has prioritized data collection from recovered COVID-19 plasma patients. Data can be inputted and accessed by all partners to facilitate further analysis [180]. The project seeks to enhance prompt feedback of outcomes from both donors (recruitment to the collection of CP) and recipients (from transfusion to clinical outcomes) [181], and outcomes are fed directly into the online database. The database holds EBA partner details and study protocols, donor and donated CP details, and recipient details and transfusion outcomes [182]. The standardization of assays at participating collection centers will support the testing and calibration of standards [183].

### 4.4. Convalescent Plasma Therapy Efficacy in Severe COVID-19 Patients

Convalescent plasma therapy shows promise in the management of a patient with severe cases of SARS-CoV-2 infection. There are no reports of major adverse effects following CP administration; however, studies/trials from which these results were obtained were poorly controlled, and the safety and efficacy of supportive care and CP therapy in patients infected with SARS-CoV-2 are not known. There is a high similarity of the receptor binding sites (RBS) between the different SARS-CoV, therefore increasing the chances of possible cross-reactivity. The SARS-CoV-specific human monoclonal antibody (CR3022) binds effectively with the COVID-19 RBS [13]. However, other SARS-CoV RBS-directed antibodies 230, 80 R, and m396 and do not bind effectively with the COVID-19 RBS [184].

CR3022 may be suggested as a possible effective l therapeutic candidate, either as a single entity or in combination with other suggested neutralizing antibodies. Recently, antibodies MAb114 and REGN-EB3 were designed and deployed in the management of Ebola virus infection, and the most interesting finding from their deployment was a drastic reduction in mortality from Ebola virus disease [185]. MAb114 and REGN-EB3 could not be deployed in the management of COVID-19 patients, considering their specificity to specific receptors. The development of a specific antibody for a virus-like SARS-CoV-2 will take a long time, therefore making the use of CP an easier option. There are recent reports of patients donating CP for SARS-CoV-2, with preliminary favorable outcomes. The observed preliminary outcomes were similar to the previously shown benefit of CP in the management of MERS, SARS, and Ebola virus infection [28]. There is presently no known international recognition of CP in the management of SARS-CoV-2 patients; however, studies are progressing in an uncontrolled case series trial. The resolution of clinical signs was observed among five critically ill patients infected with SARS-CoV-2 who were administered CP.

In this present pandemic, there are reported instances involving the use of CP in China, especially among critically ill patients infected with SARS-CoV-2 [68,69]. A pilot study was conducted among 10 critically ill COVID-19 patients in China involving the administration of CP also showed resolution of clinical signs among 10 patients who were later discharged [70]. Another case series of five critically ill COVID-19 patents in China also reported resolution of clinical signs, as shown by the decline in viral load, weaning off artificial respiration, and clinical stabilization [69]. However, sample sizes for these different studies remain a strong limitation to the studies. Clinical trials in India involving 235 patients have been associated with limited success [186]; however, there is a potential benefit that would be associated with CPT therapy especially amongst COVID-19 patients with impaired immune function due to B-cell [187]. CPT continues to be important in older adult patients with symptomatic COVID-19 [188], and more studies emphasizing the immune response in these patients would yield a further understanding and knowledge for the promotion of medicine in this field.

### 4.5. Possible Mechanisms of Action of Convalescent Plasma in COVID-19

COVID-19 is an emerging viral pandemic with severe consequences for public health, and there is no specific treatment for the diseases [159]. Passive antibody infusion is usually a short-term strategy always deployed to provide immediate immunity for individuals who are at high risk of exposure. It is always the case in the instance of a highly infectious disease outbreak. However, the deployment of CP remains a better option, especially in a case such as the SARS-CoV outbreak [189]. The therapeutic strategy emerges as the first treatment candidate for managing infectious diseases such as the current pandemic, since it has been employed successfully in other coronavirus outbreaks [159]. It is a safe and potentially effective treatment technique for emerging and re-emerging infectious agents, notably in absence of proved vaccines and antiviral agents. The intravenous immunoglobulin (IVIg) and CP have similar mechanisms of action [190].

#### 4.5.1. Antiviral Mechanisms of CP

Infusion of CP provides neutralizing antibodies (NAbs), which restrain the infection by causing viral clearance, which is required for protecting against viral illness. The concentration of NAbs in the plasma of recovered donors is correlated with the efficacy of the treatment. Using MERS and SARS-CoV models, it has been established that NAbs bind to the S1-N terminal domain, spike1-receptor binding protein (S1 RBD), and S2, which inhibits the entry and multiplication of the viruses. In addition, other antibody-mediated mechanisms such as antibody-mediated cytotoxicity, complement activation, and phagocytosis promote the therapeutic potential of CP [130,161]. Additionally, other components of plasma such as the non-neutralizing antibodies (non-NAbs) contribute to the therapeutic effect of CP. The non-NAbs including IgM and IgG bind to the virus, but in vitro studies have suggested that they do not interfere with its replication process. These are more important in facilitating the recovery of patients and in prophylaxis for infections. The role of non-NAbs in viral infections due to coronaviruses has been seen with SARS-CoV and SARS-CoV-2 where spikes of viral-specific non-NAbs (IgM and IgG) are observed during the initial stages of the infections [159,191]. In general, CP antibodies exert their antiviral effects by neutralizing the virus directly by binding to and phagocytosing it, induction of complement activation, and antibody-dependent cellular cytotoxicity (156). The antiviral properties of CP ensure that the viral load in coronaviruses is reduced [159,191]

#### 4.5.2. Immunomodulation Roles of CP

The infusion of IVIg is a critical strategy to control autoimmunity and avoid excessive innate immune cellular responses [159,191]. Similarly, CP-COVID-19 may be used to manage autoimmunity associated with COVID-19 (e.g., antiphospholipid syndrome-like disease). CP similar to IVIg prevents autoimmunity in these conditions through anti-idiotypic antibodies that blockade autoreactive antibodies (autoantibodies). The F(ab’)2 mechanisms such as blockade of autoreactive antibodies is vital in COVID-19 patients faced with the production of antiphospholipid antibodies and/or those with the antiphospholipid-like syndrome [159]. The regulation of this cascade could be critical to avoid dangerous consequences (i.e., thrombosis, disseminated intravascular coagulopathy) in these groups of patients. CP-COVID-19 neutralizes antibodies such as anti-beta2-glycoprotein, anti-cardiolipin IgA; hence, it prevents the thrombotic events [97,191]. Studies have indicated that CP, similar to IVIg has anti-inflammatory effects such as anti-inflammatory macrophage potentials, and limitation of inflammatory cascades induced by pathogenic antibodies. This inhibits cellular damage caused by complement cascade activation in excessive inflammatory environments. Various mechanisms contribute to the immunomodulation of the inflammatory reactions in COVID-19 following CP infusion through the Fc mechanism. These control excessive innate immune responses such as the control of excessive cytokine production, which prevents pulmonary damage. These immunomodulatory processes account for positive results during the management of critically ill COVID-19 patients due to treatment with CP-COVID-19. Therefore, these observations require more attention in those treated with CP, and we recommend CP administration in the early stages of the diseases to control innate immune responses and prevent lung damage [97,159]

Infusion of CP controls infection such as SARS-CoV-2 infection by direct neutralization of the virus, control of an overactive immune system through modulation of the inflammatory response, and immunomodulation of a hypercoagulable state. In addition, other plasma components of CP enhance anti-inflammatory and antiviral properties [159]. The result is a reduction of morbidity and mortality of the hospitalized patients.

## 5. Guidance on Restricted and Monitored Use of CP

Although its effectiveness and safety are mostly limited to empirical data, the ECDC, European Union competent authorities for blood and blood components, and WHO agree that CP from patients that have recovered from an infectious disease is a valuable treatment and/or pre-and post-exposure prophylactic resource for numerous infectious diseases. However, the various authorities agree that there is a need for a common approach and guidance across member states to the collection and use of CP for numerous infectious diseases [96,192]. In general, CP treatment has been increasingly adopted in clinical practice by most countries [193]. The FDA has approved the investigation of the efficacy of CP therapy for treating COVID-19 patients [194] and using convalescent plasma to treat COVID-19 [195]. In absence of proven treatments for many of the coronaviruses such as EBOV and SARS-CoV-2, many countries widely agree that CP, and other experimental interventions, should not be restricted for utilization for treating patients with these diseases. During the treatment of Ebola, any such use should be scientifically analyzed through research studies, and if CP and convalescent whole blood are to be used outside the confines of research studies, this should be considered as ‘monitored emergency use of unregistered and experimental interventions’, which was a term previously coined by the WHO during the Ebola outbreak in 2014. In the latter case, CP is used as exceptionally as an experimental intervention outside clinical trials, during cases of urgent need, and data should be collected on the efficacy and safety of these interventions [113]. Similarly, although the use of CP for treating COVID-19 patients has been approved by FDA, its use is still regulated as an investigational product. In conclusion, CP treatment has been widely adopted in clinical practice, but under the current circumstances, CP should be strictly only given in the setting of clinical trials, and countries should apply the ‘regulatory approval’ that limits its use under an ‘Emergency Use Authorization (EUA) law or in the context of clinical trials [110,194].

## 6. Access to CPT and CP Use in Low- and Middle-Income Countries

Although studies to date support the safety and efficacy of CP therapy to treat infectious diseases, CP therapy is poorly implemented, and its use is limited in the low- and middle-income countries. This is due to systemic and transfusion-specific challenges such as a low capacity for donor mobilization and collections that restrict the local procurement of the resource to meet demand [118,119]. In the African context, only Algeria, Nigeria, Namibia, Ghana, Egypt, South Africa, and few other African countries have such services and to a limited extent [118]. It is timely to promote access to CP in the low- and middle-income countries [119].

## 7. Procedure for Accessing Convalescent Plasma

### 7.1. Convalescent Plasma Collection Workflow

Using recognized collection and transfusion infrastructure, CP can be mobilized rapidly and administered. The higher the number of recovered cases, the higher the number of eligible donors of CP [196]. There are logistical challenges in procuring a reasonable inventory of CP. A workflow has been developed showing the various steps needed to be undertaken for the deployment of CP, starting from donor’s eligibility assessment, recruitment, CP collections, and transfusion. Each steps presents unique challenges.

### 7.2. Risks Associated with the Use of Convalescent Plasma

Adverse reactions have been reported from previous trials of the use of convalescent plasma during the 2015 Ebola outbreak [197]. There were reports of minor adverse reactions including increases in temperature in 5% of the patients and/or itching or skin rash in 4% of the patients [198]. There were two cases of possible transfusion-related acute lung injury (TRALI) following CP transfusion in Ebola patients [101] and another in a MERS-CoV patient [87]. In both cases of MERS-CoV and Ebola-reported adverse reactions, transfused plasma was found free of anti-HNA Ab or anti-HLA

Donors who volunteer to give plasma need to meet all relevant qualification measures for standard blood collection. In developed nations, there is one viral contamination that occurs for every 2,000,000 cases of donated plasma [199]. Negative impacts of transfused plasma additionally include “allergic transfusion reactions, transfusion-associated circulatory overload (TACO), and transfusion-related acute injury (TRALI)” [61]. The danger of a recipient getting TRALI is less than one for each 5000 donor units. TRALI is a concern for COVID-19 patients, due to the potential impacts presented by HLA on the lungs. This danger is mitigated by screening for HLA counteracting agents among female contributors [200].

Donors are not screened for normal respiratory infections. SARS-CoV-2 is not considered a transfusion infection, with only 1% of suggestive patients accounting for distinguishable SARS-CoV-2 RNA in their blood [201,202]. In the first three months of 2020, in Wuhan, 2430 blood units were screened, with only one (0.04%) contributor had SARS-CoV-2 RNA [203]. One donor (0.02%) was identified by reviewing previously screened units (4995). Potential donors will require an extra 14 days after their signs have resolved, until they are SARS-CoV-2 negative, by molecular testing [204].

Antibody-dependent enhancement (ADE) responses whereby antibodies are created during an earlier introduction to an infection can occur following the transfusion of CP. ADE is notable in Dengue infection diseases [205]. Among COVID-19 patients, ADE events can be estimated from antibodies emerging from introduction to various strains of coronaviruses. This is a potential basis in the geographic distribution of COVID-19 sickness [206]. While the effects of monoclonal antibodies (mAbs) appear to support the ADE hypothesis, the polyclonal antibodies that are found in recuperating plasma show various and different responses, yet to be clarified [207]. The nonappearance of ADE following the utilization of plasma for SARS, and MERS, is encouraging for COVID-19 patients.

### 7.3. Antibody Testing

Assurance of successful degrees of neutralizing antibodies (Abs) among COVID-19 cases is a challenge [208]. Total anti-SARS-CoV-2 and neutralizing anti-SARS-CoV-2 tests are incomparable, and it is unclear whether complete antibodies, or their components, are reasonable measures, and which antigen is best. Different types of protein molecules are used, but there are sparse data on the utilization of ELISAs for COVID-19 patients [209]. Some antibody ELISA tests showed inadequate reactivity; others reported acceptable (sensitivity about 89% and specificity about 91%) test results for IgM and IgG [210]. Seroconversion is reported to occur after 7 days, to about 20 days, from the onset of side effects [211]. In China, high titers of anti-SARS-CoV-2 antibodies are identified in the blood plasma of recovering patients, and plasma is gathered ≥14 days after the disappearance of disease [212].

### 7.4. Collection and Testing

Following donor screening, thorough tests are utilized to guarantee the wellbeing of the patient blood or blood donor [213]. Collection is frequently through apheresis, which is productive and upgrades the yield of CP (400–800 mL of plasma from apheresis contributor gives 2–4 units of CP). Units are managed in keeping standard working practice [214].

### 7.5. Distribution of Healing Plasma

Blood collection is purposeful and appropriation-dependent [215]; it is also a practice that is normally discouraged, as potential donors may not have data as regards high-hazard conduct [216]. CP is sent to hospitals for crisis management, with possible donors enlisted for crises and questions arise as to equitable distribution of units [217]. When satisfactory quantities of contributors are enrolled and high-throughput testing is accessible, this COVID-19 model will probably change [218].

### 7.6. CP Optimal Dosing and Transfusion

An investigation in China administered a solitary unit (200 mL) of plasma for post-exposure prophylaxis (PEP) [219] and suggested that 1–2 units be utilized for treatment. The viability of the immune response has been hypothesized to be from a few weeks to months [220]. Dosing was dependent on past utilization of CP treatment in SARS cases, where 5 mL/kg of plasma at a titer of 1:160 was recommended [221]. Generally, a quarter of the remedial portion is recommended for prophylaxis; 3.125 mL/kg of plasma with a titer of >1:64 was equated to an immunoglobulin level of 0.25% of 5 mL/kg plasma with a titer of 1:160. A patient who was ≈80 kg was provided 250 mL of plasma (3.125 mL/kg × 80 kg = 250 mL > 1:64), approximating the volume of a standard unit of plasma. This is yet to be worked out for pediatric patients. Appraisals of bioavailability in tissues where infections and hosts associate are yet to be established.

### 7.7. Assessment of the Safety and Efficacy of Human Anti-SARS-CoV-2 Plasma

Few clinical trials to evaluate human anti-SARS-CoV-2 plasma for the treatment of COVID-19 have been undertaken to date. A randomized, blinded phase 2 trial is ongoing to analyze the viability and safety of human anti-SARS-CoV-2 plasma versus control (SARS-CoV-2 nonimmune plasma) among those over age 18 years who have had close contact with COVID-19 [222]. The PEP presents a direct clinical advantage for those individuals at risk, including healthcare workers. A subsequent trial assessed the suitability of anti-SARS-CoV-2 plasma in patients with mild disease. The study showed that symptoms associated with stage 1 COVID were reduced, inhibition of hypoxemia in ambiance, and minimization of advancement to severity, subsequently forestalling hospitalization (common with stage 2 and 3 COVID) [223]. A third study assessed the impact of CP on patients hospitalized with coronavirus, but that did not require ICU services or utilization of assisted breath [224]. A fourth trial will assess the utilization of CP among patients requiring supported breath due to coronavirus. The safety and pharmacokinetics of CP in pediatric patients were to be analyzed in the last study since children can suffer from both serious morbidity and mortality [225,226,227]. A significant impediment to these trials is the specialized difficulties of acquiring hazard-free, plentiful amounts of CP [228]. Information on the connection between the immune response titers in the CP and viral clearance and other laboratory and clinical endpoints is key [229].

## 8. Antibodies from Recovered Coronavirus Patients Protect against COVID-19 and Strengthen the Immune System of New Patients

Antiviral medications with immunotherapy utilizing IgG could be utilized to treat or forestall coronavirus by solidifying immunity against the infection [23,230]. IgG antibodies enact the reaction through receptors on lymphocytes and other natural immune cells, and the F(ab)2 fragment acts against the antigen [28]. Fc actuates the complement system [28]. IgG from a great many benefactors establishes an IVIg [27,113,231]. The antibody-based treatment has been utilized broadly, with significant advantages detailed. For instance, it has been utilized on patients with inflammatory and autoimmune disorders, for example, idiopathic thrombocytopenic purpura, Kawasaki disease, multiple sclerosis, and lupus [82,110,112]. IVIg treatment additionally diminished intestinal irritation and infections in the gut of mice [94]. Increased development of *E. coli* and *E. faecalis* is known to cause inflammatory problems in the gastrointestinal tract [121,122]. IVIg influences the production of cytokine (72). IVIg disables microorganisms or their poisons in the gut. Likewise, it has exhibited value in the battle against parasites, fungi, and viruses [27,84,103,198]. There are some side effects of IVIg that may arise due to the nature of individual antibodies or from its preparation, but changing from IVIg to subcutaneous immunoglobulin can limit unfavorable occurrences [100,102,103]. IVIg immunomodulatory actions can lead to diminished cytokines, leukocyte adhesion molecules, Fc-gamma receptors (FcγRs), smothering pathogenic cytokine subsets, and killing pathogenic autoantibodies [107,115,116]. IVIg influences regulatory T-cells by inducing cyclo-oxygenase-2-dependent prostaglandin E2 production in dendritic cells [92].

It is critical to eliminate or inactivate any potential microbes from the plasma of recouped COVID-19 tolerant determined invulnerable IgG. Such microorganisms can be eliminated by the utilization of solvents, temperatures as high as 60 °C, and nanofiltration (20 nm) [124,232,233,234]. Terpstra et al. DATE demonstrated the use of 15 nm filtration steps, pepsin, and detergents to kill viruses from immunoglobulin [135]. By and large, immunotherapy with safe IgG joined with antiviral medications could give elective treatment against coronavirus. These resistant IgG antibodies gathered from the recuperated patients will be active against coronavirus in COVID-19 patients. Without coronavirus immunization, a blend of IgG antibodies with antiviral medications can be effective against coronavirus.

## 9. Conclusions

The emergency of SARS-CoV-2 variants necessitates the prioritization of novel therapies. CPT has led to a significant decrease in mortality rates among SARS-CoV-2 patients, although controversy on its efficacy continues to mount, demonstrating a need for further studies on immunogenicity to identify novel drug targets. Information on the ED50, EC50, and therapeutical index would help in the clinical adoption of CPT for routine medical use. Early transfusion of CP containing appropriate antibodies may inhibit the occurrence of undesired immune responses together with close monitoring of patients treated with CP to verify any unintended side effects. Antibody treatments are designed to decrease the severity of COVID-19 among patients diagnosed with the infection, and monoclonal antibody therapies from Eli Lilly and Regeneron Pharmaceuticals have recently been approved [235].

## Figures and Tables

**Table 1:** SARS-CoV-2 Variants of Concern and Variants of Interest (as of 15 June 2021).

Variants of Concern					
WHO Label	PANGO Lineage	GISAID Clade/Lineage	Next Strain Clade	Earliest Documented Samples	Date of Designation
Alpha	B.1.1.7	GRY (formerly GR/501Y.V1)	20I (V1)	United Kingdom, Sep-2020	18-Dec-2020
Beta	B.1.351	GH/501Y.V2	20H (V2)	South Africa, May-2020	18-Dec-2020
Gamma	P.1	GR/501Y.V3	20J (V3)	Brazil, Nov-2020	11-Jan-2021
Delta	B.1.617.2	G/478K.V1	21A	India, Oct-2020	VOI: 4-Apr-2021 VOC: 1
Variants of Interest					
Epsilon	B.1.427/B.1.429	GH/452R.V1	21C	United States of America, Mar-2020	5-Mar-2021
Zeta	P.2	GR/484K.V2	20B/S.484K	Brazil, Apr-2020	17-Mar-2021
Eta	B.1.525	G/484K.V3	21D	Multiple countries, Dec-2020	17-Mar-2021
Theta	P.3	GR/1092K.V1	21E	Philippines, Jan-2021	24-Mar-2021
Iota	B.1.526	GH/253G.V1	21F	United States of America, Nov-2020	24-Mar-2021
Kappa	B.1.617.1	G/452R.V3	21B	India, Oct-2020	4-Apr-2021
Lambda	C.37	GR/452Q.V1	20D	Peru, Aug-2020	14-Jun-2021

WHO = World Health Organization. GISAID = Global initiative on sharing avian flu data. PANGO = Phylogenetic Assignment of Named Global Outbreak.

## Data Availability

Data used in the study is presented within the manuscript.

## References

[1] Lai C.-C., Shih T.-P., Ko W.-C., Tang H.-J., Hsueh P.-R. (2020). Severe Acute Respiratory Syndrome Coronavirus 2 (SARS-CoV-2) and Coronavirus Disease-2019 (COVID-19): The Epidemic and the Challenges. Int. J. Antimicrob. Agents.

[2] Tyrrell D.A., Bynoe M.L. (1965). Cultivation of a Novel Type of Common-Cold Virus in Organ Cultures. Br. Med. J..

[3] Monto A.S., Cowling B., Peiris J.S.M. (2014). Coronaviruses. Viral Infect. Hum. Epidemiol. Control..

[4] Hamre D., Procknow J.J. (1966). A New Virus Isolated from the Human Respiratory Tract. Exp. Biol. Med..

[5] McIntosh K. (2004). Commentary: McIntosh K, Chao R.K., Krause H.E., Wasil R, Mocega H.E., Mufson M.A. Coronavirus Infection in Acute Lower Respiratory Tract Disease of Infants. J Infect Dis 1974; 130:502–7. J. Infect. Dis..

[6] Tyrrell D.A., Bynoe M.L., Hoorn B. (1968). Cultivation of “difficult” viruses from patients with common colds. Br. Med. J..

[7] Kahn J.S., McIntosh K. (2005). History and Recent Advances in Coronavirus Discovery. Pediatr. Infect. Dis. J..

[8] Morens D.M., Breman J.G., Calisher C.H., Doherty P.C., Hahn B.H., Keusch G.T., Kramer L.D., LeDuc J.W., Monath T.P., Taubenberger J.K. (2020). The Origin of COVID-19 and Why It Matters. Am. J. Trop. Med. Hyg..

[9] Yang Y., Peng F., Wang R., Yange M., Guan K., Jiang T., Xu G., Sun J., Chang C. (2020). The deadly coronaviruses: The 2003 SARS pandemic and the 2020 novel coronavirus epidemic in China. J. Autoimmun..

[10] Su S., Wong G., Shi W., Liu J., Lai A.C., Zhou J., Liu W., Bi Y., Gao G.F. (2016). Epidemiology, genetic recombination, and pathogenesis of coronaviruses. Trends Microbiol..

[11] Janik E., Bartos M., Niemcewicz M., Gorniak L., Bijak M. (2021). SARS-CoV-2: Outline, Prevention, and Decontamination. Pathogens.

[12] Al-Qahtani A.A. (2020). Severe Acute Respiratory Syndrome Coronavirus 2 (SARS-CoV-2): Emergence, history, basic and clinical aspects. Saudi J. Biol. Sci..

[13] Hung I.F., To K.K., Lee C.-K., Lee K.-L., Chan K., Yan W.-W., Liu R., Watt C.-L., Chan W.-M., Lai K.-Y. (2011). Convalescent plasma treatment reduced mortality in patients with severe pandemic influenza A (H1N1) 2009 virus infection. Clin. Infect. Dis..

[14] Luke T.C., Kilbane E.M., Jackson J.L., Hoffman S.L. (2006). Meta-analysis: Convalescent blood products for Spanish influenza pneumonia: A future H5N1 treatment?. Ann. Intern. Med..

[15] Xu J., Zhao S., Teng T., Abdalla A.E., Zhu W., Xie L., Wang Y., Guo X. (2020). Systematic comparison of two animal-to-human transmitted human coronaviruses: SARS-CoV-2 and SARS-CoV. Viruses.

[16] World Health Organization (2021). Middle East respiratory syndrome coronavirus (MERS-CoV). World Health Organization.

[17] Huang C., Wang Y., Li X., Ren L., Zhao J., Hu Y., Zhang L., Fan G., Xu J., Gu X. (2020). Clinical features of patients infected with 2019 novel coronavirus in Wuhan, China. Lancet.

[18] Worldometer (2021). COVID Live Update: The Coronavirus. Worldometer.

[19] Adachi S., Koma T., Doi N., Nomaguchi M., Adachi A. (2020). Commentary: Origin and evolution of pathogenic coronaviruses. Front Immunol..

[20] Mizumoto K., Kagaya K., Chowell G. (2020). Effect of a wet market on coronavirus disease (COVID-19) transmission dynamics in China, 2019–2020. Int. J. Infect. Dis..

[21] World Health Organization (2020). 2019 Novel Coronavirus (2019-nCoV) Strategic Preparedness and Response Plan for the South-East Asia Region. WHO-South East Asia..

[22] Quinlan B.D., Mou H., Zhang L., Guo Y., He W., Ojha A., Parcells M.S., Luo G., Li W., Zhong G. (2020). The SARS-CoV-2 receptor-binding domain elicits a potent neutralizing response without antibody-dependent enhancement. Immunity.

[23] Woo P.C., Lau S.K., Huang Y., Yuen K.-Y. (2009). Coronavirus diversity, phylogeny and interspecies jumping. Exp. Biol. Med..

[24] Tian S., Hu N., Lou J., Chen K., Kang X., Xiang Z., Chen H., Wang D., Liu N., Liu D. (2020). Characteristics of COVID-19 infection in Beijing. J. Infect..

[25] Baron S. (1996). Alphaviruses (Togaviridae) and Flaviviruses (Flaviviridae). Medical Microbiology.

[26] Jawhara S. (2020). Could Intravenous immunoglobulin collected from recovered coronavirus patients protect against COVID-19 and strengthen the immune system of new patients?. Int. J. Mol. Sci..

[27] Chan J.F.-W., Yuan S., Kok K.-H., To K.K.-W., Chu H., Yang J., Xing F., Liu J., Yip C.C.-Y., Poon R.W.-S. (2020). A familial cluster of pneumonia associated with the 2019 novel coronavirus indicating person-to-person transmission: A study of a family cluster. Lancet.

[28] Zhu N., Zhang D., Wang W., Li X., Yang B., Song J., Zhao X., Huang B., Shi W., Lu R. (2020). A novel coronavirus from patients with pneumonia in China, 2019. N. Engl. J. Med..

[29] Frush K., Lee G., Wald S.H., Hawn M., Krna C., Holubar M., Beatty D., Chawla A., Pinsky B.A., Schilling L. (2020). Navigating the COVID-19 Pandemic by Caring for Our Health Care Workforce as They Care for Our Patients. NEJM Catal. Innov. Care Deliv..

[30] Tang N., Li D., Wang X., Sun Z. (2020). Abnormal coagulation parameters are associated with poor prognosis in patients with novel coronavirus pneumonia. J. Thromb. Haemost..

[31] Weissman D., Alameh M.G., de Silva T., Collini P., Hornsby H., Brown R., LaBranche C.C., Edwards R.J., Sutherland L., Santra S. (2020). D614G spike mutation increases SARSCoV-2 susceptibility to neutralization. Cell Host Microbe.

[32] Mason R.J. (2020). Pathogenesis of COVID-19 from a cell biology perspective. Eur. Respir. J..

[33] Van Dorp L., Acman M., Richard D., Shaw L.P., Ford C.E., Ormond L., Owen C.J., Pang J., Tan C.C., Boshier F.A. (2020). Emergence of genomic diversity and recurrent mutations in SARS-CoV-2. Infect. Genet. Evol..

[34] Portela S.C., Brites C. (2020). Immune response in SARS-CoV-2 infection: The role of interferons type I and type III. Braz. J. Infect. Dis..

[35] Haji Abdolvahab M., Moradi-Kalbolandi S., Zarei M., Bose D., Majidzadeh-A K., Farahmand L. (2021). Potential role of interferons in treating COVID-19 patients. Int. Immunopharmacol..

[36] Wang X., Che Q., Ji X., Meng X., Zhang L., Jia R., Lyu H., Bai W., Tan L., Gao Y. (2021). Correlation between lung infection severity and clinical laboratory indicators in patients with COVID-19: A cross-sectional study based on machine learning. BMC Infect. Dis..

[37] Carsana L., Sonzogni A., Nasr A., Rossi R.S., Pellegrinelli A., Zerbi P., Rech R., Colombo R., Antinori S., Corbellino M. (2021). Pulmonary post-mortem findings in a series of COVID-19 cases from northern Italy: A two-centre descriptive study. Lancet. Infect. Dis..

[38] Cevik M., Kuppalli K., Kindrachuk J., Peiris M. (2020). Virology, transmission, and pathogenesis of SARS CoV-2. BMJ.

[39] Chatterjee S.K., Saha S., Munoz M.N.M. (2020). Molecular Pathogenesis, Immunopathogenesis and Novel Therapeutic Strategy Against COVID-19. Front Mol. Biosci..

[40] Konings F., Perkins M.D., Kuhn J.H., Pallen M.J., Alm E.J., Archer B.N., Barakat A., Bedford T., Bhiman J.N., Caly L. (2021). SARS-CoV-2 Variants of Interest and Concern naming scheme conducive for global discourse. Nat. Microbiol..

[41] WHO (2021). Tracking SARS-CoV-2 Variants. World Health Organization.

[42] Lippi G., Henry B.M. (2021). How will emerging SARS-CoV-2 variants impact herd immunity?. Ann. Transl. Med..

[43] Rambaut A., Holmes E.C., O’Toole Á., Hill V., McCrone J.T., Ruis C., du Plessis L., Pybus O.G. (2020). A dynamic nomenclature proposal for SARS-CoV-2 to assist genomic epidemiology. Nat. Microbiol..

[44] Giovanetti M., Benvenuto D., Angeletti S., Ciccozzi M. (2020). The first two cases of 2019-nCoV in Italy: Where they come from?. J. Med. Virol..

[45] Zhao Z., Li H., Wu X., Zhong Y., Zhang K., Zhang Y.P., Boerwinkle E., Fu Y.X. (2004). Moderate mutation rate in the SARS coronavirus genome and its implications. BMC Evol. Biol..

[46] Dudas G., Carvalho L.M., Rambaut A., Bedford T. (2018). MERS-CoV spillover at the camel-human interface. eLife.

[47] Snijder E.J., Bredenbeek P.J., Dobbe J.C., Thiel V., Ziebuhr J., Poon L.L., Guan Y., Rozanov M., Spaan W.J., Gorbalenya A.E. (2003). Unique and conserved features of genome and proteome of SARS-coronavirus, an early split-off from the coronavirus group 2 lineage. J. Mol. Biol..

[48] Minskaia E., Hertzig T., Gorbalenya A.E., Campanacci V., Cambillau C., Canard B., Ziebuhr J. (2006). Discovery of an RNA virus 3‘ > 5‘ exo-ribonuclease that is critically involved in coronavirus RNA synthesis. Proc. Natl Acad. Sci. USA.

[49] Lythgoe K.A., Hall M., Ferretti L., de Cesare M., MacIntyre-Cockett G., Trebes A., Andersson M., Otecko N., Wise E.L., Moore N. (2020). Shared SARS-CoV-2 diversity suggests localised transmission of minority variants. bioRxiv.

[50] Arora P., Pöhlmann S., Hoffmann M. (2021). Mutation D614G increases SARS-CoV-2 transmission. Signal Transduct. Target. Ther..

[51] Volz E., Hill V., McCrone J.T., Price A., Jorgensen D., O’Toole Á., Southgate J., Johnson R., Jackson B., Nascimento F.F. (2021). Evaluating the Effects of SARS-CoV-2 Spike Mutation D614G on Transmissibility and Pathogenicity. Cell.

[52] Milewska A., Kindler E., Vkovski P., Zeglen S., Ochman M., Thiel V., Rajfur Z., Pyrc K. (2018). APOBEC3-mediated restriction of RNA virus replication. Sci. Rep..

[53] Naveca F.G., Nascimento V., de Souza V.C., Corado A.D.L., Nascimento F., Silva G., Costa Á., Duarte D., Pessoa K., Mejía M. (2021). COVID-19 in Amazonas, Brazil, was driven by the persistence of endemic lineages and P.1 emergence. Nat. Med..

[54] Sabino E.C., Buss L.F., Carvalho M., Prete C.A., Crispim M., Fraiji N.A., Pereira R., Parag K.V., da Silva Peixoto P., Kraemer M. (2021). Resurgence of COVID-19 in Manaus, Brazil, despite High Sero-prevalence. Lancet.

[55] Woo P.C.Y., Wong B.H., Huang Y., Lau S.K., Yuen K.-Y. (2007). Cytosine deamination and selection of CpG suppressed clones are the two major independent biological forces that shape codon usage bias in coronaviruses. Virology.

[56] Rice A.M., Morales A.C., Ho A.T., Mordstein C., Mühlhausen S., Watson S., Cano L., Young B., Kudla G., Hurst L.D. (2021). Evidence for Strong Mutation Bias toward, and Selection against, U Content in SARS-CoV-2: Implications for Vaccine Design. Mol. Biol. Evol..

[57] Di Giorgio S., Martignano F., Torcia M.G., Mattiuz G., Conticello S.G. (2020). Evidence for host-dependent RNA editing in the transcriptome of SARS-CoV-2. Sci. Adv..

[58] Simmonds P. (2020). Rampant C→U hypermutation in the genomes of SARS-CoV-2 and other coronaviruses: Causes and consequences for their short- and longterm evolutionary trajectories. mSphere.

[59] Domingo-Calap P., Schubert B., Joly M., Solis M., Untrau M., Carapito R., Georgel P., Caillard S., Fafi-Kremer S., Paul N. (2018). An unusually high substitution rate in transplant-associated BK polyomavirus in vivo is further concentrated in HLA-C-bound viral peptides. PLoS Pathog..

[60] Greaney A.J., Loes A.N., Crawford K., Starr T.N., Malone K.D., Chu H.Y., Bloom J.D. (2021). Comprehensive mapping of mutations in the SARS-CoV-2 receptor-binding domain that affect recognition by polyclonal human plasma antibodies. Cell Host Microbe.

[61] Yang X., Yu Y., Xu J., Shu H., Liu H., Wu Y., Zhang L., Yu Z., Fang M., Yu T. (2020). Clinical course and outcomes of critically ill patients with SARS-CoV-2 pneumonia in Wuhan, China: A single-centered, retrospective, observational study. Lancet Respir. Med..

[62] Li L., Sun W., Han M., Ying Y., Wang Q. (2020). A Study on the Predictors of Disease Severity of COVID-19. Med. Sci. Monit. Int. Med. J. Exp. Clin. Res..

[63] Chen N., Zhou M., Dong X., Qu J., Gong F., Han Y., Qiu Y., Wang J., Liu Y., Wei Y. (2020). Epidemiological and clinical characteristics of 99 cases of 2019 novel coronavirus pneumonia in Wuhan, China: A descriptive study. Lancet.

[64] Wu J., Li W., Shi X., Chen Z., Jiang B., Liu J., Wang D., Liu C., Meng Y., Cui L. (2020). Early antiviral treatment contributes to alleviate the severity and improve the prognosis of patients with novel coronavirus disease (COVID-19). J. Intern. Med..

[65] Levi M., Thachil J., Iba T., Levy J.H. (2020). Coagulation abnormalities and thrombosis in patients with COVID-19. Lancet Haematol..

[66] Wichmann D., Sperhake J.-P., Lütgehetmann M., Steurer S., Edler C., Heinemann A., Heinrich F., Mushumba H., Kniep I., Schröder A.S. (2020). Autopsy Findings and Venous Thromboembolism in Patients With COVID-19. Ann. Intern. Med..

[67] Iba T., Levy J.H., Connors J.M., Warkentin T.E., Thachil J., Levi M. (2020). The unique characteristics of COVID-19 coagulopathy. Crit. Care.

[68] Munster V.J., Koopmans M., van Doremalen N., van Riel D., de Wit E. (2020). A novel coronavirus emerging in China—key questions for impact assessment. N. Engl. J. Med..

[69] Wang C., Horby P.W., Hayden F.G., Gao G.F. (2020). A novel coronavirus outbreak of global health concern. Lancet.

[70] Guan W.-j., Ni Z.-y., Hu Y., Liang W.-h., Ou C.-q., He J.-x., Liu L., Shan H., Lei C.-l., Hui D.S. (2020). Clinical characteristics of coronavirus disease 2019 in China. N. Engl. J. Med..

[71] Thachil J., Tang N., Gando S., Falanga A., Cattaneo M., Levi M., Clark C., Iba T. (2020). ISTH interim guidance on recognition and management of coagulopathy in COVID-19. J. Thromb. Haemost..

[72] Klekotka R.B., Mizgała E., Król W. (2015). The etiology of lower respiratory tract infections in people with diabetes. Pneumonol. Alergol. Pol..

[73] MacLaren G., Fisher D., Brodie D. (2020). Preparing for the Most Critically Ill Patients With COVID-19: The Potential Role of Extracorporeal Membrane Oxygenation. JAMA.

[74] Gao Y.d., Ding M., Dong X., Zhang J.j., Kursat Azkur A., Azkur D., Gan H., Sun Y.l., Fu W., Li W. (2021). Risk factors for severe and critically ill COVID-19 patients: A review. Allergy.

[75] Duan K., Liu B., Li C., Zhang H., Yu T., Qu J., Zhou M., Chen L., Meng S., Hu Y. (2020). Effectiveness of convalescent plasma therapy in severe COVID-19 patients. Proc. Natl. Acad. Sci. USA.

[76] Guo L., Ren L., Yang S., Xiao M., Chang D., Yang F., Dela Cruz C.S., Wang Y., Wu C., Xiao Y. (2020). Profiling early humoral response to diagnose novel coronavirus disease (COVID-19). Clin. Infect. Dis..

[77] Zhou 2020 C., Bu G., Sun Y., Ren C., Qu M., Gao Y., Zhu Y., Wang L., Sun L., Liu Y. (2020). Evaluation of serum IgM and IgG antibodies in COVID-19 patients by enzyme linked immunosorbent assay. J. Med. Virol..

[78] Bradfute S.B., Hurwitz I., Yingling A.V., Ye C., Cheng Q., Noonan T.P., Raval J.S., Sosa N.R., Mertz G.J., Perkins D.J. (2020). Severe Acute Respiratory Syndrome Coronavirus 2 Neutralizing Antibody Titers in Convalescent Plasma and Recipients in New Mexico: An Open Treatment Study in Patients with Coronavirus Disease 2019. J. Infect. Dis..

[79] Pau A.K., Aberg J., Baker J., Belperio P.S., Coopersmith C., Crew P., Glidden D.V., Grund B., Gulick R.M., Harrison C. (2021). Convalescent plasma for the treatment of COVID-19: Perspectives of the National Institutes of Health COVID-19 Treatment Guidelines Panel. Ann. Intern. Med..

[80] Dieterle M.E., Haslwanter D., Bortz III R.H., Wirchnianski A.S., Lasso G., Vergnolle O., Abbasi S.A., Fels J.M., Laudermilch E., Florez C. (2020). A replication-competent vesicular stomatitis virus for studies of SARS-CoV-2 spike-mediated cell entry and its inhibition. bioRxiv.

[81] Joyner M.J., Bruno K.A., Klassen S.A., Kunze K.L., Johnson P.W., Lesser E.R., Wiggins C.C., Senefeld J.W., Klompas A.M., Hodge D.O. (2020). Safety update: COVID-19 convalescent plasma in 20,000 hospitalized patients. Mayo Clin. Proc..

[82] Feldmann H., Geisbert T.W. (2011). Ebola haemorrhagic fever. Lancet.

[83] Kilgore P.E., Grabenstein J.D., Salim A.M., Rybak M. (2015). Treatment of Ebola virus disease. Pharmacother. J. Hum. Pharmacol. Drug Ther..

[84] Torjesen I. (2021). COVID-19 vaccine shortages: What is the cause and what are the implications?. BMJ.

[85] Shen C., Wang Z., Zhao F., Yang Y., Li J., Yuan J., Wang F., Li D., Yang M., Xing L. (2020). Treatment of 5 critically ill patients with COVID-19 with convalescent plasma. JAMA.

[86] Rubin R. (2020). Testing an Old Therapy against a New Disease: Convalescent Plasma for COVID-19. JAMA.

[87] Wiley S.R. (2019). Adaptive immunity profiling and methods for generation of monoclonal antibodies. U.S. Patent.

[88] Samad N., Sodunke T.E., Al Banna H., Sapkota A., Fatema A.N., Iskandar K., Jahan D., Hardcastle T.C., Nusrat T., Chowdhury T.S. (2020). Convalescent Plasma Therapy for Management of COVID-19: Perspectives and Deployment in the Current Global Pandemic. Risk Manag. Healthc. Policy.

[89] Franchini M., Liumbruno G.M. (2021). Convalescent Plasma for the Treatment of Severe COVID-19. Biologics Targets Ther..

[90] Garraud O. (2020). Passive immunotherapy with convalescent plasma against COVID-19? What about the evidence base and clinical trials?. Transfus. Apher. Sci..

[91] Shankar-Hari M., Estcourt L., Harvala H., Roberts D., Menon D.K. (2020). Convalescent plasma to treat critically ill patients with COVID-19: Framing the need for randomised clinical trials. Crit. Care.

[92] De Oliveira F.A., Nucci M.P., Rego G.N.D.A., Alves A.D.H., Marti L.C., Nucci L.P., Mamani J.B., Gamarra L.F. (2021). Convalescent plasma therapy in COVID-19 critically ill patients during advanced phases of clinical trials and their preliminary results. Einstein.

[93] Chaplin D.D. (2010). Overview of the immune response. J. Allergy Clin. Immunol..

[94] Bloch E.M., Goel R., Montemayor C., Cohn C., Tobian A.A. (2021). Promoting access to COVID-19 convalescent plasma in low- and middle-income countries. Transfus. Apher. Sci..

[95] Mahmood A., Mahmood R., Khan M., Ali S., Khan N., Jaffar S.R. (2021). Convalescent plasma therapy for Covid 19—A perspective. Hematol. Transfus. Int. J..

[96] WHO (2015). Ethics of Using Convalescent Whole Blood and Convalescent Plasma during the Ebola Epidemic. World Health Organization. https://apps.who.int/iris/handle/10665/161912.

[97] Nagoba B., Gavkare A., Jamadar N., Mumbre S., Selkar S. (2020). Positive aspects, negative aspects and limitations of plasma therapy with special reference to COVID-19. J. Infect. Public Heal..

[98] Zhao Q., He Y. (2020). Challenges of Convalescent Plasma Therapy on COVID-19. J. Clin. Virol..

[99] Rejeki M.S., Sarnadi N., Wihastuti R., Fazharyasti V., Samin W.Y., Yudhaputri F.A., Johar E., Nurainy N., Bachtiar N.S., Muljono D.H. (2021). Convalescent plasma therapy in patients with moderate-to-severe COVID-19: A study from Indonesia for clinical research in low- and middle-income countries. EClinicalMedicine.

[100] Wang K.Y., Shah P., Pierce M. (2021). Convalescent plasma for COVID-19 complicated by ARDS due to TRALI. BMJ Case Rep..

[101] Vial P.A., Valdivieso F., Calvo M., Rioseco M.L., Riquelme R., Araneda A., Tomicic V., Graf J., Paredes L., Florenzano M. (2014). A nonrandomized multicentre trial of human immune plasma for treatment of hantavirus cardiopulmonary syndrome by ANDV. Antivir. Ther..

[102] Qiu X., Wong G., Audet J., Bello A., Fernando L., Alimonti J.B., Fausther-Bovendo H., Wei H., Aviles J., Hiatt E. (2014). Reversion of advanced Ebola virus disease in nonhuman primates with ZMapp. Nature.

[103] Zhang Y., Li D., Jin X., Huang Z. (2014). Fighting Ebola with ZMapp: Spotlight on plant-made antibody. Sci. China Life Sci..

[104] Lyon G.M., Mehta A.K., Varkey J.B., Brantly K., Plyler L., McElroy A.K., Kraft C.S., Towner J.S., Spiropoulou C., Ströher U. (2014). Clinical care of two patients with Ebola virus disease in the United States. N. Engl. J. Med..

[105] Flexner S., Lewis P.A. (1910). Experimental Poliomyelitis in Monkeys: Seventh Note: Active Immunization and Passive Serum Protection. J. Am. Med. Assoc..

[106] Pettitt J., Zeitlin L., Kim D.H., Working C., Johnson J.C., Bohorov O., Bratcher B., Hiatt E., Hume S.D., Johnson A.K. (2013). Therapeutic intervention of Ebola virus infection in rhesus macaques with the MB-003 monoclonal antibody cocktail. Sci. Transl. Med..

[107] Murin C.D., Fusco M.L., Bornholdt Z.A., Qiu X., Olinger G.G., Zeitlin L., Kobinger G.P., Ward A.B., Saphire E.O. (2014). Structures of protective antibodies reveal sites of vulnerability on Ebola virus. Proc. Natl. Acad. Sci. USA.

[108] Tsang T., Lai-Yin T., Pak-Yin L., Lee M. (2003). Update: Outbreak of severe acute respiratory syndrome-worldwide, 2003. MMWR Morb. Mortal. Wkly. Rep..

[109] Ng E.K., Ng P.-C., Hon K.E., Cheng W.F., Hung E.C., Chan K.A., Chiu R.W., Li A.M., Poon L.L., Hui D.S. (2003). Serial analysis of the plasma concentration of SARS coronavirus RNA in pediatric patients with severe acute respiratory syndrome. Clin. Chem..

[110] Zhao Z., Zhang F., Xu M., Huang K., Zhong W., Cai W., Yin Z., Huang S., Deng Z., Wei M. (2003). Description and clinical treatment of an early outbreak of severe acute respiratory syndrome (SARS) in Guangzhou, PR China. J. Med. Microbiol..

[111] Mailles A., Blanckaert K., Chaud P., Van der Werf S., Lina B., Caro V., Campese C., Guéry B., Prouvost H., Lemaire X. (2013). First cases of Middle East Respiratory Syndrome Coronavirus (MERS-CoV) infections in France, investigations and implications for the prevention of human-to-human transmission, France, May 2013. Eurosurveillance.

[112] Arabi Y.M., Balkhy H.H., Hayden F.G., Bouchama A., Luke T., Baillie J.K., Al-Omari A., Hajeer A.H., Senga M., Denison M.R. (2017). Middle East respiratory syndrome. N. Engl. J. Med..

[113] Cheke R.S., Shinde S., Ambhore J., Adhao V., Cheke D. (2020). Coronavirus: Hotspot on coronavirus disease 2019 in India. Indian J. Med. Sci..

[114] Pullano G., Pinotti F., Valdano E., Boëlle P.-Y., Poletto C., Colizza V. (2020). Novel coronavirus (2019-nCoV) early-stage importation risk to Europe, January 2020. Eurosurveillance.

[115] Amoss H.L., Chesney A.M. (1917). A report on the serum treatment of twenty-six cases of epidemic poliomyelitis. J. Exp. Med..

[116] McGuire L., Redden W. (1918). The Use of Convalescent Human Serum in Influenza Pneumonia—A Preliminary Report. Am. J. Public Health.

[117] Nabarro D., Signy A. (1931). Convalescent serum in prophylaxis of measles. Br. Med. J..

[118] Leider J.P., Brunker P.A., Ness P.M. (2010). Convalescent transfusion for pandemic influenza: Preparing blood banks for a new plasma product?. Transfusion.

[119] Cheng Y., Wong R., Soo Y., Wong W., Lee C., Ng M., Chan P., Wong K., Leung C., Cheng G. (2005). Use of convalescent plasma therapy in SARS patients in Hong Kong. Eur. J. Clin. Microbiol. Infect. Dis..

[120] Mair-Jenkins J., Saavedra-Campos M., Baillie J.K., Cleary P., Khaw F.-M., Lim W.S., Makki S., Rooney K.D., Group C.P.S., Nguyen-Van-Tam J.S. (2015). The effectiveness of convalescent plasma and hyperimmune immunoglobulin for the treatment of severe acute respiratory infections of viral etiology: A systematic review and exploratory meta-analysis. J. Infect. Dis..

[121] World Health Organization (1978). Ebola haemorrhagic fever in Zaire, 1976. Report of an international commission. Bull World Health Organ.

[122] Marshall Lyon G., Mehta A.K., Ribner B.S. (2017). Clinical Management of Patients with Ebola Virus Disease in High-Resource Settings. Curr. Top. Microbiol. Immunol..

[123] Emond R., Evans B., Bowen E., Lloyd G. (1977). A case of Ebola virus infection. Br. Med. J..

[124] Mora-Rillo M., Arsuaga M., Ramírez-Olivencia G., de la Calle F., Borobia A.M., Sánchez-Seco P., Lago M., Figueira J.C., Fernández-Puntero B., Viejo A. (2015). Acute respiratory distress syndrome after convalescent plasma use: Treatment of a patient with Ebola virus disease contracted in Madrid, Spain. Lancet Respir. Med..

[125] Gupta M., Mahanty S., Bray M., Ahmed R., Rollin P.E. (2001). Passive transfer of antibodies protects immunocompetent and immunodeficient mice against lethal Ebola virus infection without complete inhibition of viral replication. J. Virol..

[126] Baize S., Leroy E.M., Georges-Courbot M.-C., Capron M., Lansoud-Soukate J., Debré P., Fisher-Hoch S.P., McCormick J.B., Georges A.J. (1999). Defective humoral responses and extensive intravascular apoptosis are associated with fatal outcome in Ebola virus-infected patients. Nat. Med..

[127] World Health Organization *Use of Convalescent Whole Blood or Plasma Collected from Patients Recovered from Ebola Virus Disease for Transfusion, as an Empirical Treatment during Outbreaks: Interim Guidance for National Health Authorities and Blood Transfusion Services*. Version 1.0. September 2014. World Health Organization. https://apps.who.int/iris/handle/10665/135591.

[128] Liu G., Wong G., Su S., Bi Y., Plummer F., Gao G.F., Kobinger G., Qiu X. (2017). Clinical Evaluation of Ebola Virus Disease Therapeutics. Trends Mol. Med..

[129] Kraft C.S., Hewlett A.L., Koepsell S., Winkler A.M., Kratochvil C.J., Larson L., Varkey J.B., Mehta A.K., Lyon III G.M., Friedman-Moraco R.J. (2015). The use of TKM-100802 and convalescent plasma in 2 patients with Ebola virus disease in the United States. Clin. Infect. Dis..

[130] Van Griensven J., De Weiggheleire A., Delamou A., Smith P.G., Edwards T., Vandekerckhove P., Bah E.I., Colebunders R., Herve I., Lazaygues C. (2016). The Use of Ebola Convalescent Plasma to Treat Ebola Virus Disease in Resource-Constrained Settings: A Perspective from the Field. Clin. Infect. Dis..

[131] AlQahtani M., Abdulrahman A., Almadani A., Alali S.Y., Al Zamrooni A.M., Hejab A.H., Conroy R.M., Wasif P., Otoom S., Atkin S.L. (2021). Randomized controlled trial of convalescent plasma therapy against standard therapy in patients with severe COVID-19 disease. Sci. Rep..

[132] Liddell A.M., Davey R.T., Mehta A.K., Varkey J.B., Kraft C.S., Tseggay G.K., Badidi O., Faust A.C., Brown K.V., Suffredini A.F. (2015). Characteristics and clinical management of a cluster of 3 patients with Ebola virus disease, including the first domestically acquired cases in the United States. Ann. Intern. Med..

[133] Zaki A.M., Van Boheemen S., Bestebroer T.M., Osterhaus A.D., Fouchier R.A. (2012). Isolation of a novel coronavirus from a man with pneumonia in Saudi Arabia. N. Engl. J. Med..

[134] World Health Organization (2015). Guidelines on Assessing Donor Suitability for Blood Donation.

[135] Zumla A., Hui D.S., Perlman S. (2015). Middle East respiratory syndrome. Lancet.

[136] Choe P.G., Perera R., Park W.B., Song K.H., Bang J.H., Kim E.S., Kim H.B., Ko L., Park S.W., Kim N.J. (2017). MERS-CoV Antibody Responses 1 Year after Symptom Onset, South Korea, 2015. Emerg. Infect. Dis..

[137] Hung I.F., To K.K., Lee C.-K., Lee K.-L., Yan W.-W., Chan K., Chan W.-M., Ngai C.-W., Law K.-I., Chow F.-L. (2013). Hyperimmune IV immunoglobulin treatment: A multicenter double-blind randomized controlled trial for patients with severe 2009 influenza A (H1N1) infection. Chest.

[138] Letko M., Munster V. (2020). Functional assessment of cell entry and receptor usage for lineage B β-coronaviruses, including 2019-nCoV. BioRxiv.

[139] Tang X., Wu C., Li X., Song Y., Yao X., Wu X., Duan Y., Zhang H., Wang Y., Qian Z. (2020). On the origin and continuing evolution of SARS-CoV-2. Natl. Sci. Rev..

[140] RECOVERY Collaborative Group (2020). Lopinavir-ritonavir in patients admitted to hospital with COVID-19 (RECOVERY): A randomised, controlled, open-label, platform trial. Lancet.

[141] Funk C.D., Laferrière C., Ardakani A. (2020). A Snapshot of the Global Race for Vaccines Targeting SARS-CoV-2 and the COVID-19 Pandemic. Front. Pharmacol..

[142] Locht C. (2020). Vaccines against COVID-19. Anaesth. Crit. Care Pain Med..

[143] Lindberg D. (2000). Internet access to the National Library of Medicine. Eff. Clin. Pract..

[144] Richardson T., Johnston A.M., Draper H. (2017). A systematic review of Ebola treatment trials to assess the extent to which they adhere to ethical guidelines. PLoS ONE.

[145] Jahrling P.B., Geisbert J.B., Swearengen J.R., Larsen T., Geisbert T.W. (2007). Ebola hemorrhagic fever: Evaluation of passive immunotherapy in nonhuman primates. J. Infect. Dis..

[146] Bloch E.M. (2020). Convalescent plasma to treat COVID-19. J. Am. Soc. Hematol..

[147] Murphy M., Estcourt L., Grant-Casey J., Dzik S. (2020). International survey of trials of convalescent plasma to treat COVID-19 infection. Transfus. Med. Rev..

[148] To K.K.-W., Tsang O.T.-Y., Leung W.-S., Tam A.R., Wu T.-C., Lung D.C., Yip C.C.-Y., Cai J.-P., Chan J.M.-C., Chik T.S.-H. (2020). Temporal profiles of viral load in posterior oropharyngeal saliva samples and serum antibody responses during infection by SARS-CoV-2: An observational cohort study. Lancet Infect. Dis..

[149] Zhao J., Yuan Q., Wang H., Liu W., Liao X., Su Y., Wang X., Yuan J., Li T., Li J. (2020). Antibody responses to SARS-CoV-2 in patients of novel coronavirus disease 2019. Clin. Infect. Dis..

[150] Davey Jr R.T., Fernández-Cruz E., Markowitz N., Pett S., Babiker A.G., Wentworth D., Khurana S., Engen N., Gordin F., Jain M.K. (2019). Anti-influenza hyperimmune intravenous immunoglobulin for adults with influenza A or B infection (FLU-IVIG): A double-blind, randomised, placebo-controlled trial. Lancet Respir. Med..

[151] Rodriguez W.J., Gruber W.C., Groothuis J.R., Simoes E.A., Rosas A.J., Lepow M., Hemming V., Group R.-I.S. (1997). Respiratory syncytial virus immune globulin treatment of RSV lower respiratory tract infection in previously healthy children. Pediatrics.

[152] Dzik S. (2020). COVID-19 Convalescent Plasma: Now Is the Time for Better Science. Transfus. Med. Rev..

[153] Iacob S., Iacob D.G. (2020). SARS-CoV-2 Treatment Approaches: Numerous Options, No Certainty for a Versatile Virus. Front. Pharmacol..

[154] Marano G., Vaglio S., Pupella S., Facco G., Catalano L., Liumbruno G.M., Grazzini G. (2016). Convalescent plasma: New evidence for an old therapeutic tool?. Blood Transfus..

[155] Burnouf T., Seghatchian J. (2014). Ebola virus convalescent blood products: Where we are now and where we may need to go. Transfus. Apher. Sci..

[156] Shimoni Z., Niven M.J., Pitlick S., Bulvik S. (2001). Treatment of West Nile virus encephalitis with intravenous immunoglobulin. Emerg. Infect. Dis..

[157] Haley M., Retter A.S., Fowler D., Gea-Banacloche J., O’Grady N.P. (2003). The role for intravenous immunoglobulin in the treatment of West Nile virus encephalitis. Clin. Infect. Dis..

[158] Rojas M., Monsalve D.M., Pacheco Y., Acosta-Ampudia Y., Ramírez-Santana C., Ansari A.A., Gershwin M.E., Anaya J.-M. (2020). Ebola virus disease: An emerging and re-emerging viral threat. J. Autoimmun..

[159] Rojas M., Rodríguez Y., Monsalve D.M., Acosta-Ampudia Y., Camacho B., Gallo J.E., Rojas-Villarraga A., Ramírez-Santana C., Díaz-Coronado J.C., Manrique R. (2020). Convalescent plasma in COVID-19: Possible mechanisms of action. Autoimmun. Rev..

[160] Sharun K., Tiwari R., Iqbal Yatoo M., Patel S.K., Natesan S., Dhama J., Malik Y.S., Harapan H., Singh R.K., Dhama K. (2020). Antibody-based immunotherapeutics and use of convalescent plasma to counter COVID-19: Advances and prospects. Expert Opin. Biol. Ther..

[161] Casadevall A., Pirofski L.-a. (2020). The convalescent sera option for containing COVID-19. J. Clin. Investig..

[162] Ye M., Fu D., Ren Y., Wang F., Wang D., Zhang F., Xia X., Lv T. (2020). Treatment with convalescent plasma for COVID-19 patients in Wuhan, China. J. Med. Virol..

[163] Chang L., Zhao L., Gong H., Wang L., Wang L. (2020). Severe Acute Respiratory Syndrome Coronavirus 2 RNA Detected in Blood Donations. Emerg. Infect. Dis..

[164] Chan A.P., Yeung J.F., Yu C.C., Wang S.Q., Ke Y. (2011). Empirical study of risk assessment and allocation of public-private partnership projects in China. J. Manag. Eng..

[165] Garraud O., Heshmati F., Pozzetto B., Lefrere F., Girot R., Saillol A., Laperche S. (2016). Plasma therapy against infectious pathogens, as of yesterday, today and tomorrow. Transfus. Clin. Biol..

[166] Lünemann J.D., Nimmerjahn F., Dalakas M.C. (2015). Intravenous immunoglobulin in neurology—mode of action and clinical efficacy. Nat. Rev. Neurol..

[167] Gonaglea D., Sharifa K., O’Regand A., Bridgewood C. (2020). The Role of Cytokines including Interleukin-6 in COVID-19 induced Pneumonia and Macrophage Activation Syndrome-Like Disease. Autoimmun. Rev..

[168] Wan S., Yi Q., Fan S., Lv J., Zhang X., Guo L., Lang C., Xiao Q., Xiao K., Yi Z. (2020). Characteristics of lymphocyte subsets and cytokines in peripheral blood of 123 hospitalized patients with 2019 novel coronavirus pneumonia (NCP). MedRxiv.

[169] Sahr F., Ansumana R., Massaquoi T., Idriss B., Sesay F., Lamin J., Baker S., Nicol S., Conton B., Johnson W. (2017). Evaluation of convalescent whole blood for treating Ebola Virus Disease in Freetown, Sierra Leone. J. Infect..

[170] Zhou B., Zhong N., Guan Y. (2007). Treatment with convalescent plasma for influenza A (H5N1) infection. N. Engl. J. Med..

[171] Wu X.-X., Gao H.-N., Wu H.-B., Peng X.-M., Ou H.-L., Li L.-J. (2015). Successful treatment of avian-origin influenza A (H7N9) infection using convalescent plasma. Int. J. Infect. Dis..

[172] Taylor P.C., Adams A.C., Hufford M.M., de la Torre I., Winthrop K., Gottlieb R.L. (2021). Neutralizing monoclonal antibodies for treatment of COVID-19. Nat. Rev. Immunol..

[173] Lotz M.T., Moore M.L., Peebles R.S. (2013). Respiratory syncytial virus and reactive airway disease. Curr. Top. Microbiol. Immunol..

[174] Luke T.C., Casadevall A., Watowich S.J., Hoffman S.L., Beigel J.H., Burgess T.H. (2010). Hark back: Passive immunotherapy for influenza and other serious infections. Crit. Care Med..

[175] Gajic O., Rana R., Winters J.L., Yilmaz M., Mendez J.L., Rickman O.B., O’Byrne M.M., Evenson L.K., Malinchoc M., DeGoey S.R. (2007). Transfusion-related acute lung injury in the critically ill: Prospective nested case-control study. Am. J. Respir. Crit. Care Med..

[176] Wan Y., Shang J., Sun S., Tai W., Chen J., Geng Q., He L., Chen Y., Wu J., Shi Z. (2020). Molecular mechanism for antibody-dependent enhancement of coronavirus entry. J. Virol..

[177] Brown B.L., McCullough J. (2020). Treatment for emerging viruses: Convalescent plasma and COVID-19. Transfus. Apher. Sci..

[178] Crowe J.E., Firestone C.-Y., Murphy B.R. (2001). Passively acquired antibodies suppress humoral but not cell-mediated immunity in mice immunized with live attenuated respiratory syncytial virus vaccines. J. Immunol..

[179] Mulangu S., Dodd L.E., Davey Jr R.T., Tshiani Mbaya O., Proschan M., Mukadi D., Lusakibanza Manzo M., Nzolo D., Tshomba Oloma A., Ibanda A. (2019). A randomized, controlled trial of Ebola virus disease therapeutics. N. Engl. J. Med..

[180] Nagurney A., Dutta P. (2021). A Multiclass, Multiproduct COVID-19 Convalescent Plasma Donor Equilibrium Model. SN Oper. Res. Forum.

[181] World Health Organization (2021). Blood Donor Selection: Guidelines on Assessing Donor Suitability for Blood Donation.

[182] Sundström B., Johansson I., Rantapää-Dahlqvist S. (2015). Interaction between dietary sodium and smoking increases the risk for rheumatoid arthritis: Results from a nested case–control study. Rheumatology.

[183] Geisen C., Kann G., Strecker T., Wolf T., Schüttfort G., van Kraaij M., MacLennan S., Rummler S., Weinigel C., Eickmann M. (2016). Pathogen-reduced Ebola virus convalescent plasma: First steps towards standardization of manufacturing and quality control including assessment of Ebola-specific neutralizing antibodies. Vox Sang..

[184] Gordon C.J., Tchesnokov E.P., Feng J.Y., Porter D.P., Götte M. (2020). The antiviral compound remdesivir potently inhibits RNA-dependent RNA polymerase from Middle East respiratory syndrome coronavirus. J. Biol. Chem..

[185] Winkler A.M., Koepsell S.A. (2015). The use of convalescent plasma to treat emerging infectious diseases: Focus on Ebola virus disease. Curr. Opin. Hematol..

[186] Agarwal A., Mukherjee A., Kumar G., Chatterjee P., Bhatnagar T., Malhotra P. (2020). Convalescent plasma in the management of moderate COVID-19 in adults in India: Open label phase II multicentre randomised controlled trial (PLACID Trial). BMJ.

[187] Rodionov R.N., Biener A., Spieth P., Achleitner M., Hölig K., Aringer M., Mingrone G., Corman V.M., Drosten C., Bornstein S.R. (2021). Potential benefit of convalescent plasma transfusions in immunocompromised patients with COVID-19. Lancet Microbe.

[188] Katz L.M. (2021). (A Little) Clarity on Convalescent Plasma for COVID-19. N. Engl. J. Med..

[189] Cheng M.P., Papenburg J., Desjardins M., Kanjilal S., Quach C., Libman M., Dittrich S., Yansouni C.P. (2020). Diagnostic testing for severe acute respiratory syndrome–related coronavirus-2: A narrative review. Ann. Intern. Med..

[190] Ouyang J., Isnard S., Lin J., Fombuena B., Peng X., Routy J.-P., Chen Y. (2020). Convalescent Plasma: The Relay Baton in the Race for Coronavirus Disease 2019 Treatment. Front. Immunol..

[191] Sanders R.W., Derking R., Cupo A., Julien J.-P., Yasmeen A., de Val N., Kim H.J., Blattner C., de la Peña A.T., Korzun J. (2013). A next-generation cleaved, soluble HIV-1 Env trimer, BG505 SOSIP. 664 gp140, expresses multiple epitopes for broadly neutralizing but not non-neutralizing antibodies. PLoS Pathog..

[192] European Comission (2020). An EU Programme of COVID-19 Convalescent Plasma Collection and Transfusion-Guidance on Collection, Testing, Processing, Storage, Distribution and Monitored Use. https://www.google.com/url?sa=t&source=web&cd=&ved=2ahUKEwj1-tiyu6XxAhVjx4UKHTw8BPkQFjAAegQIAxAF&url=https%3A%2F%2Fec.europa.eu%2Fhealth%2Fsites%2Fdefault%2Ffiles%2Fblood_tissues_organs%2Fdocs%2Fguidance_plasma_covid19_en.pdf&usg=AOvVaw0SNoqWZDT2ETPcHFhqmbKt.

[193] Kanj S., Al-Omari B. (2021). Convalescent Plasma Transfusion for the Treatment of COVID-19 in Adults: A Global Perspective. Viruses.

[194] Estcourt L.J., Roberts D.J. (2020). Convalescent plasma for COVID-19. BMJ.

[195] Oreh A.C. (2020). Is COVID-19 Convalescent Plasma an option for Africa?. Afr. Sang..

[196] Zhang L., Pang R., Xue X., Bao J., Ye S., Dai Y., Zheng Y., Fu Q., Hu Z., Yi Y. (2020). Anti-SARS-CoV-2 virus antibody levels in convalescent plasma of six donors who have recovered from COVID-19. Aging.

[197] Henao-Restrepo A.M., Longini I.M., Egger M., Dean N.E., Edmunds W.J., Camacho A., Carroll M.W., Doumbia M., Draguez B., Duraffour S. (2015). Efficacy and effectiveness of an rVSV-vectored vaccine expressing Ebola surface glycoprotein: Interim results from the Guinea ring vaccination cluster-randomised trial. Lancet.

[198] Zhang A.P., Abelson D.M., Bornholdt Z.A., Liu T., Woods J., Virgil L and Saphire E.O. (2012). The ebolavirus VP24 interferon antagonist: Know your enemy. Virulence.

[199] Cicchetti A., Berrino A., Casini M., Codella P., Facco G., Fiore A., Marano G., Marchetti M., Midolo E., Minacori R. (2016). Health Technology Assessment of pathogen reduction technologies applied to plasma for clinical use. Blood Transfus..

[200] Cho M.S., Modi P., Sharma S. (2021). Transfusion-related Acute Lung Injury. [Updated 2021 May 7]. StatPearls [Internet].

[201] Sayyed A.F. (2020). The Corona (COVID-19) (SARS-CoV-2) cure using Blood Transfusion and Machine learning. IRE J..

[202] Shaw A.M., Hyde C., Merrick B., James-Pemberton P., Squires B.K., Olkhov R.V., Batra R., Patel A., Bisnauthsing K., Nebbia G. (2020). Real-world evaluation of a novel technology for quantitative simultaneous antibody detection against multiple SARS-CoV-2 antigens in a cohort of patients presenting with COVID-19 syndrome. Analyst.

[203] Fakhar Z., Khan S., AlOmar S.Y., Alkhuriji A., Ahmad A. (2021). ABBV-744 as a potential inhibitor of SARS-CoV-2 main protease enzyme against COVID-19. Sci. Rep..

[204] Malik Y.S., Kumar N., Sircar S., Kaushik R., Bhatt S., Dhama K., Gupta P., Goyal K., Singh M.P., Ghoshal U. (2020). Pandemic Coronavirus Disease (COVID-19): Challenges and A Global Perspective. Pathogens.

[205] Saiz J.-C., Martín-Acebes M.A., Bueno-Marí R., Salomón O.D., Villamil-Jiménez L.C., Heukelbach J., Alencar C.H., Armstrong P.K., Ortiga-Carvalho T.M., Mendez-Otero R. (2017). Zika virus: What have we learnt since the start of the recent epidemic?. Front. Microbiol..

[206] Tetro J.A. (2020). Is COVID-19 receiving ADE from other coronaviruses?. Microbes Infect..

[207] Korber B., Fischer W.M., Gnanakaran S., Yoon H., Theiler J., Abfalterer W., Hengartner N., Giorgi E.E., Bhattacharya T., Foley B. (2020). Tracking changes in SARS-CoV-2 Spike: Evidence that D614G increases infectivity of the COVID-19 virus. Cell.

[208] Bhalla N., Pan Y., Yang Z., Payam A.F. (2020). Opportunities and challenges for biosensors and nanoscale analytical tools for pandemics: COVID-19. ACS Nano.

[209] Boettler T., Marjot T., Newsome P.N., Mondelli M.U., Maticic M., Cordero E., Jalan R., Moreau R., Cornberg M., Berg T. (2020). Impact of COVID-19 on the care of patients with liver disease: EASL-ESCMID position paper after 6 months of the pandemic. JHEP Rep..

[210] Elshal M.F., McCoy J.P. (2006). Multiplex bead array assays: Performance evaluation and comparison of sensitivity to ELISA. Methods.

[211] Sekirov I., Russell S.L., Antunes L.C.M., Finlay B.B. (2010). Gut microbiota in health and disease. Physiol. Rev..

[212] Wang X., Guo X., Xin Q., Pan Y., Hu Y., Li J., Chu Y., Feng Y., Wang Q. (2020). Neutralizing antibodies responses to SARS-CoV-2 in COVID-19 inpatients and convalescent patients. Clin. Infect. Dis..

[213] Fridey J.L., Townsend M.J., Kessler D.A., Gregory K.R. (2007). A question of clarity: Redesigning the American Association of Blood Banks blood donor history questionnaire—a chronology and model for donor screening. Transfus. Med. Rev..

[214] Mokrzycki M.H., Balogun R.A. (2011). Therapeutic apheresis: A review of complications and recommendations for prevention and management. J. Clin. Apher..

[215] Storni C. (2010). Multiple forms of appropriation in self-monitoring technology: Reflections on the role of evaluation in future self-care. Int. J. Hum. Comput. Interact..

[216] Copeman J. (2005). Veinglory: Exploring processes of blood transfer between persons. J. R. Anthropol. Inst..

[217] Power K., McCrea Z., White M., Breen A., Dunleavy B., O’Donoghue S., Jacquemard T., Lambert V., El-Naggar H., Delanty N. (2020). The development of an epilepsy electronic patient portal: Facilitating both patient empowerment and remote clinician-patient interaction in a post-COVID-19 world. Epilepsia.

[218] Batra R., Chan H., Kamath G., Ramprasad R., Cherukara M.J., Sankaranarayanan S.K. (2020). Screening of therapeutic agents for COVID-19 using machine learning and ensemble docking studies. J. Phys. Chem. Lett..

[219] Diemunsch P., Grélot L. (2000). Potential of substance P antagonists as antiemetics. Drugs.

[220] Wood G.J., Bughi S., Morrison J., Tanavoli S., Tanavoli S., Zadeh H.H. (2003). Hypnosis, differential expression of cytokines by T-cell subsets, and the hypothalamo-pituitary-adrenal axis. Am. J. Clin. Hypn..

[221] Lee W.S., Wheatley A.K., Kent S.J., DeKosky B.J. (2020). Antibody-dependent enhancement and SARS-CoV-2 vaccines and therapies. Nat. Microbiol..

[222] Gouda A.S., Mégarbane B. (2020). Snake venom-derived bradykinin-potentiating peptides: A promising therapy for COVID-19?. Drug Dev. Res..

[223] Long Q.-X., Tang X.-J., Shi Q.-L., Li Q., Deng H.-J., Yuan J., Hu J.-L., Xu W., Zhang Y., Lv F.-J. (2020). Clinical and immunological assessment of asymptomatic SARS-CoV-2 infections. Nat. Med..

[224] Sterne J.A., Murthy S., Diaz J.V., Slutsky A.S., Villar J., Angus D.C., Annane D., Azevedo L.C.P., Berwanger O., Cavalcanti A.B. (2020). Association between administration of systemic corticosteroids and mortality among critically ill patients with COVID-19: A meta-analysis. JAMA.

[225] Bourgeois F.T., Murthy S., Pinto C., Olson K.L., Ioannidis J.P., Mandl K.D. (2012). Pediatric versus adult drug trials for conditions with high pediatric disease burden. Pediatrics.

[226] Alifano M., Alifano P., Forgez P., Iannelli A. (2020). Renin-angiotensin system at the heart of COVID-19 pandemic. Biochimie.

[227] Ramireddy A., Chugh H., Reinier K., Ebinger J., Park E., Thompson M., Cingolani E., Cheng S., Marban E., Albert C.M. (2020). Experience with Hydroxychloroquine and Azithromycin in the COVID-19 Pandemic: Implications for QT Interval Monitoring. J. Am. Heart Assoc..

[228] Ward P., Small I., Smith J., Suter P., Dutkowski R. (2005). Oseltamivir (Tamiflu^®^) and its potential for use in the event of an influenza pandemic. J. Antimicrob. Chemother..

[229] Tricou V., Minh N.N., Van T.P., Lee S.J., Farrar J., Wills B., Tran H.T., Simmons C.P. (2010). A randomized controlled trial of chloroquine for the treatment of dengue in Vietnamese adults. PLoS Negl. Trop. Dis..

[230] Tyrrell D.A., Myint S.H. (1996). Coronaviruses. Medical Microbiology.

[231] Kumar D., Malviya R., Sharma P.K. (2020). Corona virus: A review of COVID-19. Eurasian J. Med. Oncol..

[232] Johnson K., Webb P., Heymann D. (1978). Evaluation of the Plasmapheresis Program in Zaire. Ebola Virus Hemorrhagic Fever.

[233] Mupapa K., Massamba M., Kibadi K., Kuvula K., Bwaka A., Kipasa M., Colebunders R., Muyembe-Tamfum J. (1999). Treatment of Ebola hemorrhagic fever with blood transfusions from convalescent patients. J. Infect. Dis..

[234] Buijs P.R., Verhagen J.H., van Eijck C.H., van den Hoogen B.G. (2015). Oncolytic viruses: From bench to bedside with a focus on safety. Hum. Vaccines Immunother..

[235] Reuters Italy Approves GSK-Vir Antibody to Treat COVID-19. Published on 13 July 2021. https://www.reuters.com/world/europe/italy-approves-gsk-vir-antibody-treat-covid-19-2021-07-13/.

